# Biocompatible Polyelectrolyte Complex Nanoparticles for Lycopene Encapsulation Attenuate Oxidative Stress-Induced Cell Damage

**DOI:** 10.3389/fnut.2022.902208

**Published:** 2022-05-27

**Authors:** Dongjing Zhang, Yun Jiang, Ming Xiang, Fen Wu, Min Sun, XianFeng Du, Lei Chen

**Affiliations:** ^1^Anhui Key Laboratory of Eco-Engineering and Biotechnology, School of Life Sciences, Anhui University, Hefei, China; ^2^School of Biological and Food Engineering, Suzhou University, Suzhou, China; ^3^State Key Laboratory of Tea Plant Biology and Utilization, Anhui Agricultural University, Hefei, China

**Keywords:** lycopene, PEC NPs, storage stability, sustained release, antioxidant capacity

## Abstract

In this study, lycopene was successfully encapsulated in polyelectrolyte complex nanoparticles (PEC NPs) fabricated with a negatively charged polysaccharide, TLH-3, and a positively charged sodium caseinate (SC) *via* electrostatic interactions. Results showed that the lycopene-loaded PEC NPs were spherical in shape, have a particle size of 241 nm, have a zeta potential of −23.6 mV, and have encapsulation efficiency of 93.6%. Thus, lycopene-loaded PEC NPs could serve as effective lycopene carriers which affected the physicochemical characteristics of the encapsulated lycopene and improved its water dispersibility, storage stability, antioxidant capacity, and sustained release ability in aqueous environments when compared with the free lycopene. Moreover, encapsulated lycopene could enhance the cells' viability, prevent cell apoptosis, and protect cells from oxidative damage through the Nrf2/*HO-1*/AKT signalling pathway, *via* upregulation of antioxidase activities and downregulation of MDA and ROS levels. Therefore, the biocompatible lycopene-loaded PEC NPs have considerable potential use for the encapsulation of hydrophobic nutraceuticals in the food and pharmaceutical industries.

## Introduction

Lycopene is a carotenoid primarily found in ripe tomatoes and carrots (1), and it has been widely used as a colourant, an antioxidant, and a flavouring agent in the food industry. Lycopene, as a functional ingredient, has attracted considerable academic attention because of its antioxidant, anti-inflammatory, and antitumor effects on humans, and it can reduce the risk of prostate cancer and cardiovascular disease (2). However, lycopene is a hydrophobic compound with low water-solubility, gastrointestinal instability, high photosensitivity, thermal sensitivity, and inferior bioavailability, which limits its practical application in functional foods and health care products (3). Thus, an effective delivery system is necessary to improve lycopene absorption and bioavailability. Researchers have made substantial attempts to enhance the bioavailability and functionality of lycopene by using various delivery carriers or vehicles such as nanocomplexes, emulsions, nanoemulsions, polymeric micelles, liposomes, and nanostructured lipid carriers (NLCs) (4–9). Polyelectrolyte complexes that are based on oppositely charged polysaccharides and proteins seem to be ideal microcarriers, which can improve the water solubility, stability, compatibility, and bioavailability of lycopene (10). In recent years, the usage of PE complexation and biomolecular delivery via liposomes and lipid nanoparticles has received attention, particularly from the viewpoint of ES-driven complexation. Cherstvy (11) presented the exact solution of the linear Poisson–Boltzmann equation for several problems relevant to electrostatics of DNA complexes with cationic lipids. Caetano (12) investigated the adsorption properties of hen egg-white lysozyme into a negatively charged silica pore by using a coarse-grained model and using constant-pH Monte Carlo simulations.

Casein is an easily digestible protein derived from milk, and it has been extensively utilised in the food industry as a flavouring agenet, a colourant, and a preservative. The properties, including remarkable surface activity, stability, emulsification, and self-assembly, promote the combining capacity with ions and small molecules and facilitate their functionality in drug delivery systems (13, 14). In addition, casein can improve the bioavailability of bioactive substances because of its shielding capabilities, which are essential for protecting sensitive payloads. Casein can polymerise with polyanions through electrostatic interaction to form nanoparticle polymers, which can be used as carrier materials for the transport of bioactive substances (10). We used sodium caseinate (SC) which exists in the form of protein particles through self-assembly in an aqueous solution with a diameter of 10–20 nm, which has good emulsification, thermal stability, film formation, and rheological properties (15). Compared with other food proteins, SC can form a thick sterically stabilising layer on the emulsion droplet interface that protects newly formed droplets against flocculation and coalescence (16). Thus, encapsulating bioactive compounds into SC nanoparticles can improve water dispersibility and physicochemical stability. Tricholoma lobayense, a delicious edible mushroom, has been commonly utilised as a functional food in China and other Asian nations for its taste and health values (17). Polysaccharide TLH-3 isolated from Tricholoma lobayense is an acidic polysaccharide with a molecular weight of 4.22 × 10^3^ Da, which is primarily composed of 1,3-linked-α-d-glucopyranosyl branched at C-6 and 1,3-linked-β-d-galactopyranosyl (18). Our previous studies have proven that polysaccharide TLH-3 exerts excellent antioxidant, anti-ageing, tumour-suppressing, and immunoregulatory abilities (17, 19). TLH-3 exists in an aqueous solution in the form of negatively charged polyanions and forming natural polymer polysaccharide hydrosol, which has good water solubility, emulsification, and stability (18). Thus, TLH-3 can form stable polyelectrolyte complex nanoparticles (PEC NPs) with polyanions such as SC in aqueous media for lycopene encapsulation. However, to our best knowledge, no studies have been conducted on the construction and utilisation of PEC via polyanion SC and polysaccharide TLH-3 for lycopene to improve solubility, bioactivity, and bioavailability of lycopene.

At present, considerable evidence suggests that, when oxidation and antioxidants are uneven in the body, the elevated production of reactive oxygen species (ROS) causes disruptions in the normal mechanism involved in cellular signalling pathways, leading to cellular dysfunction and apoptotic cell death (20, 21). The pivotal signal transduction pathway, such as the PI3K/AKT pathway *in vivo*, is involved in multiple cellular processes such as cell growth and survival induced by oxidative stress, and it regulates the expression of various inflammatory mediators and cytokines (22, 23). Studies have found that chronic diseases such as cancer, diabetes, cardiovascular disease, and neurodegenerative diseases (24) are related to oxidative stress, and ameliorating oxidative stress has become an effective way to alleviate these chronic diseases. Therefore, finding drugs with strong antioxidant activity is important to relieve various diseases caused by oxidative stress.

In this study, we aimed to construct stable PEC NPs with negatively charged TLH-3 and positively charged SC in an aqueous solution. Lycopene was incorporated into PEC NPs, and the storage stability, controlled release, and antioxidant activity of lycopene in PEC NPs were evaluated. Moreover, the protective effect of lycopene-loaded PEC NPs on H_2_O_2_-induced cellular oxidative damage and the underlying mechanism were also investigated for the first time. Thus, lycopene-loaded PEC NPs might constitute a potential health supplement or pharmaceutical product to improve human health and well-being in the food and pharmaceutical industries.

## Materials and Methods

### Materials and Chemicals

Lycopene (with a purity of ≥98%, product codes: 820354) and sodium caseinate (with a purity of ≥98.07%, product codes: Z3625) were purchased from Sigma Corporation (St Louis, MO, USA). Polysaccharide TLH-3 was prepared according to the method described in our previous study (16). The degree of branching (DB) value and weight-average molecular weight (Mw) were 0.74 and 4.22 × 103 g/mol, respectively. Human normal hepatocytes (L02 cells) were purchased from the Shanghai Institutes for Biological Sciences, Chinese Academy of Sciences (Shanghai, China). 2, 4, 6-tris (2-pyridyl)-s-triazine, 2-diphenyl-1-picrylhydrazyl (DPPH), and 2, 2-azinobis (3-ethylbenzothiazoline-6-sulphonic acid) (ABTS) were purchased from Sigma-Aldrich Chemical Co. (St. Louis, MO, USA). All primary and secondary antibodies were purchased from Abcam (Abcam, Cambridge, UK). All other chemicals and solvents were of an analytical grade or higher and were obtained from commercial sources.

### Preparation of PEC NPs and Lycopene-Loaded PEC NPs

Sodium caseinate and TLH-3 were dissolved in deionised water and magnetically stirred overnight to completely hydrate SC and TLH-3 powders. Then, the obtained solutions were passed through a 0.45 μm filtration membrane to prepare stock solutions of SC and TLH-3 (10 mg/ml). The TLH-3 solution (10 mg/ml) was added to the SC solution (10 mg/ml) and magnetically stirred at various mass ratios (TLH-3:SC = 1:10, 2:10, 3:10, 4:10 w/w). The pH was adjusted to the desired value (2, 3, 4, 5, 6, and 7) by adding an aqueous solution containing 0.1 mol/L of HCl and/or 1 mol/L of NaOH. The obtained TLH-3/SC mixture was lyophilised to yield the final PEC NPs after the solution was finally subjected to ultrasonic treatment for 1 h. The effects of the TLH-3/SC mass ratio and pH on PEC NP formation were studied and recorded.

**Figure 1:**
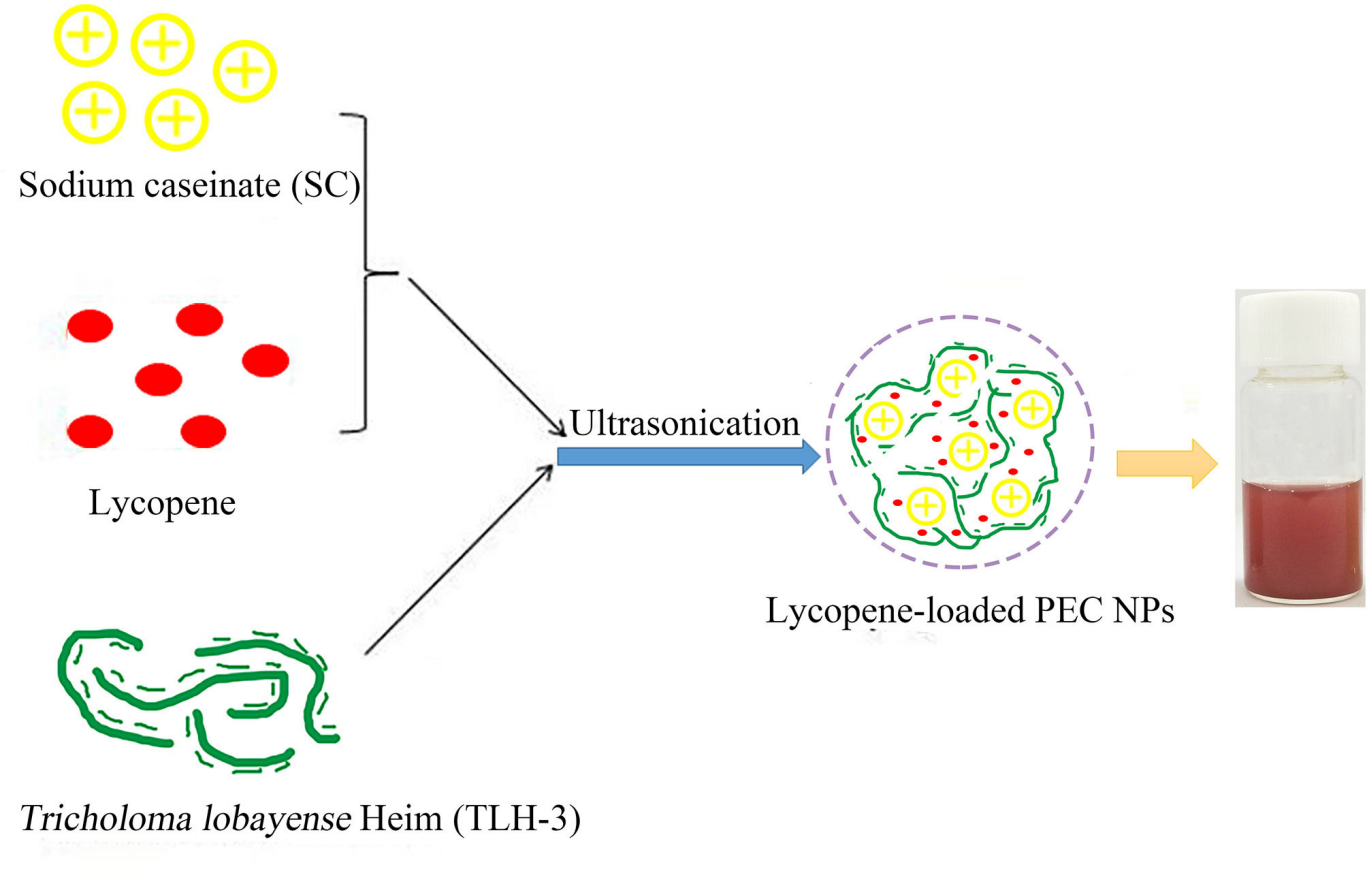
The detailed schematic for the production of lycopene-loaded polyelectrolyte complex nanoparticles (PEC NPs).

Lycopene was added to the SC solution in different proportions (4, 8, 12, and 16% W/W). After stirring and mixing, the solution was ultrasonically treated for 1 h. The pH value was adjusted to 3 with 1 mol/L of HCl. Then, TLH-3 solution (10 mg/ml) was added to a mixed solution at various mass ratios (1:10, 2:10, 3:10, and 4:10 w/w) and then magnetically stirred. Free lycopene was removed by centrifugation (5,000 rpm, 15 min), and the final lycopene-loaded PEC NPs were obtained by freeze-drying. The detailed schematic for the production of lycopene-loaded PEC NPs is shown in Figure 1.

### Characterisation of PEC NPs and Lycopene-Loaded PEC NPs

Polyelectrolyte NPs and lycopene-loaded PEC NPs were diluted with deionised water. Then, the zeta potential, Z-average size, and polydispersity index of PEC NPs and lycopene-loaded PEC NPs were measured by using a Zetasizer dynamic light scattering detector and Zeta Siser Nano Series (Malvern, United Kingdom). All measurements were performed at least three times at 25°C, and the results were averaged. Fourier transform infrared (FT-IR) spectroscopy was used to characterise the chemical structure of lycopene, SC, TLH-3, PEC NPs, and lycopene-loaded PEC NP samples. The dried samples were ground with potassium bromide powder and pressed into a pellet for spectrometric measurement. FT-IR spectroscopy was performed using a VERTEX 80 FT-IR spectrometer (Bruker Co., Ettlingen, Germany) within the wavelength range of 400–4,000 cm^−1^. Differential scanning calorimetry (DSC) thermograms of lycopene, PEC NPs, and lycopene-loaded PEC NPs were measured using a DSC Q2000 thermal analyser (TA Co., USA). Five milligrammes of samples were placed in aluminium capsules and sealed with aluminium lids. Subsequently, thermal analysis was performed in a dry nitrogen atmosphere with a flow rate of 50 ml·min^−1^, and the temperature was increased from 25 to 300°C at a heating rate of 10°C·min^−1^. The crystalline and amorphous nature of lycopene, PEC NPs, and lycopene-loaded PEC NPs were analysed by X-ray diffraction (XRD) (SmartLab 9KW, JEOL, Co., Japan). XRD patterns were recorded at 45 kV and 200 mA within the 2θ range of 5–50° at a scanning rate of 4°·min^−1^. Thermogravimetric analysis of lycopene, PEC NPs, and lycopene-loaded PEC NP samples (5 mg) was performed on a thermogravimetric analyser (TG 209F3, Netzsch, Germany) at a heating rate of 20°C·min^−1^ under nitrogen with a mass flow rate of 40 mL·min^−1^ at 40 to 800°C. A transmission electron microscope (TEM; JEM-1400flash, JEOL, 120 kV) was used to observe the morphology and particle size of PEC NPs and lycopene-loaded PEC NPs. For TEM detection, the samples were diluted with ultra-pure water and then dripped onto copper wires to dry before testing.

### Encapsulation Efficiency (EE) and Loading Content (LC) of Lycopene

**Figure 2:**
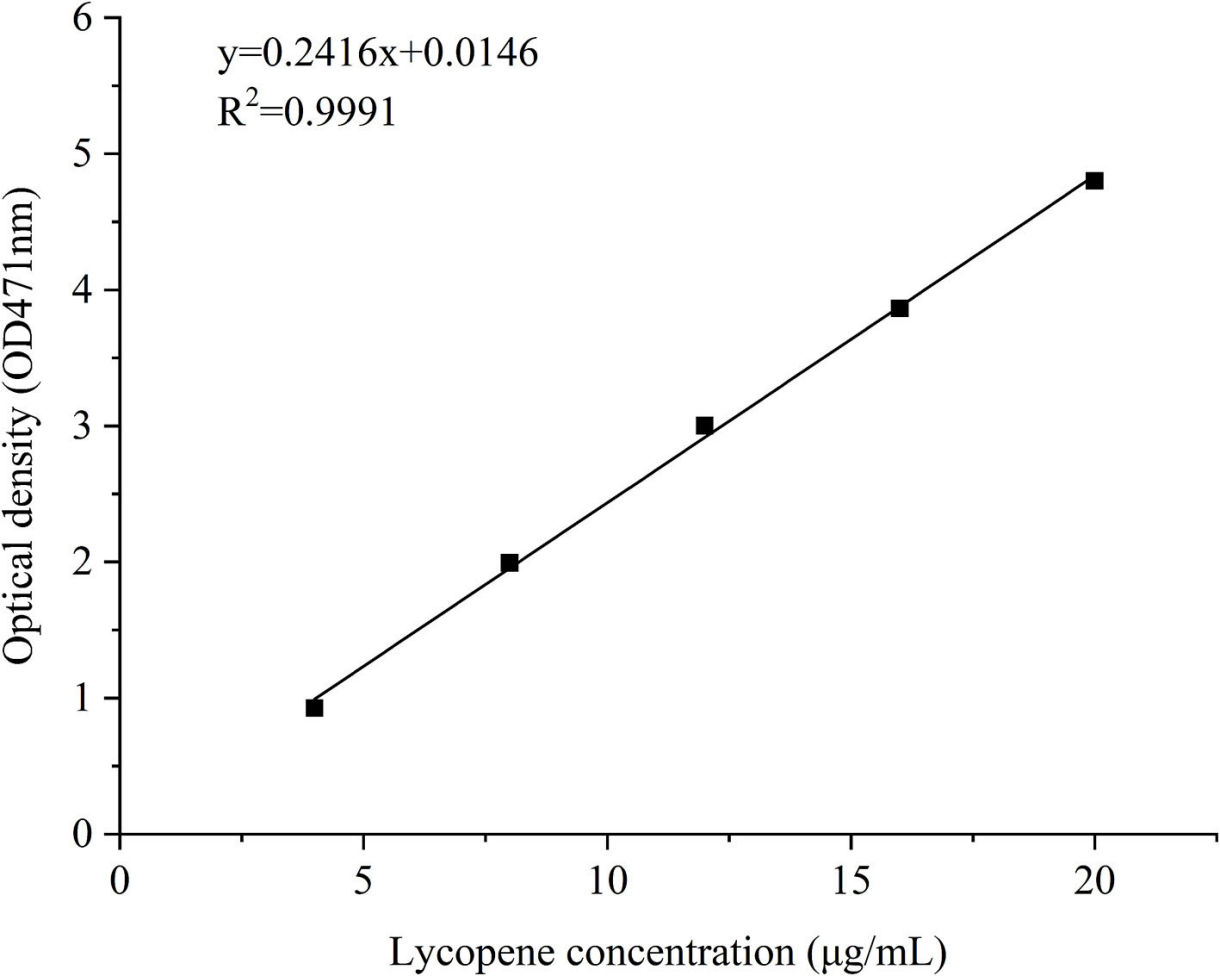
A standard curve of free lycopene.

Polyelectrolyte NP samples were prepared in accordance with Method 2.3, and freshly prepared lycopene-loaded PEC NPs were centrifuged at 10,000 rpm for 10 min to precipitate unencapsulated lycopene. The supernatant was collected, and ethyl acetate was added to the precipitate until it was completely dissolved. The concentration of lycopene in the collected supernatant was determined by spectrophotometry at a wavelength of 471 nm using a standard curve of free lycopene (y = 0.2461x + 0.0146, *R*^2^ = 0.9991) (Figure 2). The EE and LC of lycopene were determined as described previously with minor modifications (25).

All experiments were repeated three times at room temperature. Calculations were performed in accordance with the following equations:


(1) $$\begin{array}{rcl} EE\;\left(\%\right)\; & = & \;\frac{Lycopene\;encapsulated\;in\;PEC\;NPs}{Total\;weight\;of\;lycopene}\;\times \;100 \end{array}$$

(2) $$\begin{array}{rcl} LC\;\left(\%\right)\; & = & \;\frac{Lycopene\;encapsulated\;in\;PEC\;NPs}{Total\;weight\;of\;lycopene-loaded\;PEC\;NPs}\;\times \;100 \end{array}$$

### Storage Stability

The storage stability of lycopene and lycopene-loaded PEC NPs at different temperatures and the light was estimated. The thermal stability of lycopene and lycopene-loaded PEC NPs was estimated in accordance with previous studies with slight modifications (25, 26). In brief, the solutions of lycopene and lycopene-loaded PEC NPs were stored in a sealed glass bottle under dark conditions and then incubated at 0, 25, 45, 65, and 85°C for 0.5 h in a thermostatic water bath. All samples were equilibrated at room temperature for 10 min before analysis. The light stability of lycopene and lycopene-loaded PEC NPs was estimated using a light incubator in accordance with a previous study (27). In brief, the solutions of lycopene and lycopene-loaded PEC NPs were placed in a light incubator and exposed to a lamp (10 W) for different durations (0, 5, 10, 15, and 20 days) at room temperature. Afterwards, the resultant solutions of all tests were centrifuged at 5,000 × g for 10 min. The collected supernatants were diluted and spectrophotometrically analysed at 471 nm. The residual concentration of lycopene in PEC NPs was derived from the standard curve of lycopene (Figure 2). The retention rate of lycopene was measured using the following equation in accordance with a previously published report (28):


(3) $$\begin{array}{l} \begin{array}{c} Retention\;rate\;of\;lycopene\;(\%)= \end{array}\\ \begin{array}{c} \;\frac{the\;content\;of\;lycopene\;after\;test}{the\;initial\;content\;of\;lycopene}\;\times \;100 \end{array} \end{array}$$

### *In vitro* Drug Release Studies

The drug release profiles of lycopene-loaded PEC NPs and free lycopene in the simulated gastrointestinal fluid were assessed in accordance with a published report with minor modifications (29). In brief, 10 ml of freshly prepared lycopene-loaded PEC NPs (10 mg/ml) and free lycopene were mixed with 10 ml of simulated gastric fluid (SGF, pH 1.5, containing 0.5 g of NaCl, 2 ml of HCl and 0.8 g of pepsin added in 250 ml of deionised water), and the resulting pH was adjusted to three by adding 0.1 mol/L of HCl. The mixed dispersions were then conducted in a thermostatic shaker (37°C, 100 rpm/min) under gentle shaking for 2 h. Samples were removed at 0.5, 1, 1.5, and 2 h for analysis. Afterwards, gastric digestion was terminated by adjusting the pH of mixed dispersions to 7. Subsequently, the resulting gastric digestion dispersions were mixed with simulated intestinal fluid (SIF, pH 7.5, containing 1.7 g of KH_2_PO_4_, 0.38 g of NaOH, 2.5 g of pancreatin, and 1.25 g of bile salts added in 250 ml of deionised water). After intestinal digestion, the samples were incubated for 4 h at 37°C in a thermostatic shaker at 100 rpm. Samples were collected at 0.5, 1, 1.5, 2, 2.5, 3, 3.5, 4, 5, and 6 h for analysis. The released lycopene of collected samples at SGF and SIF digestion stages was determined spectrophotometrically at 471 nm, as described in Section 2.4. Lycopene release (%) was quantified using the following equation:


(4) $$\begin{array}{r} Lycopene\;release\;\left(\%\right)=\frac{\;Released\;lycopene}{Total\;lycopene}\;\times \;100 \end{array}$$

### *In vitro* Antioxidant Activity Analysis

*In vitro* antioxidant activities of lycopene in acetone, lycopene in water, and lycopene-loaded PEC NPs was evaluated by DPPH radical scavenging ability, hydroxyl radical scavenging ability, and ABTS radical scavenging ability. Solutions of lycopene in acetone, lycopene in water, and lycopene-loaded PEC NPs at different concentrations (0–100 μg/ml) were added with the same volume of DPPH reagent separately. Then, DPPH radical scavenging activities were evaluated as described previously with minor modifications (30). Hydroxyl radical scavenging activities were measured in accordance with a previously published report (31). In addition, ABTS was determined based on a previously described method with modifications (30). The absorbance of the resulting solution was determined using a TU-190 spectrophotometer (Beijing Puxi General Analytical Instrument Co., Ltd., China). For all assays, vitamin C (Vc) was used as the positive control, and distilled water was used as the blank control.

The scavenging effects were all calculated in accordance with the following equation:


(5) $$\begin{array}{r} Scavenging\;activity\;\left(\%\right)\;=\;\frac{A_{0}-A_{1}}{A_{0}}\;\times \;100 \end{array}$$

where *A*_0_ denotes the absorbance value of a blank control in the system, and *A*_1_ denotes the absorbance value of different samples.

### Protective Effects of Lycopene-Loaded PEC NPs Against Cellular Stress Damage

#### Cell Culture and Treatment

L02 cells were cultured in DMEM in accordance with a previously described method (32). Cells at the sub-confluence level were subjected to various concentrations of H_2_O_2_ (0, 100, 200, 400, 600, and 800 μM) in fresh and DMEM high-glucose medium for 4 h to induce oxidative damage until cell apoptosis with condensed and fragmented nuclei. Various concentrations of H_2_O_2_ treatments were selected to determine the appropriate H_2_O_2_ concentration for cell apoptosis induction by detecting cell viability.

#### Cell Viability Assay

Cell viability was measured using MTT assays as described by Wang et al. (33). L02 cells were seeded into 96-well-plates at a density of 1 × 10^5^/mL in a culture medium overnight and then treated with different concentrations of lycopene-loaded PEC NP solution (1, 5, 10, and 25 μmol/L) for 24 h. MTT solution was subsequently added to each well, and absorbance was measured at 570 nm using a microplate reader to determine whether lycopene-loaded PEC NPs have a cytotoxic effect on L02 cells. Next, L02 cells were treated with the most appropriate concentration of H_2_O_2_ solution for 4 h to set up a cell oxidative damage model to evaluate the protection of lycopene-loaded PEC NP solution against H_2_O_2_-induced cell apoptosis. Then, cells were washed two times with PBS (pH 7.4) and treated with various concentrations of lycopene-loaded PEC NP solution (1, 5, and 10 μmol/L) for 24 h. MTT solution (.5 mg/ml, 100 μL) was added to each well and incubated at 37°C for an additional 4 h. The absorbance value was detected at 570 nm using a microtitre plate reader (Bio-Rad, California, USA). Cell viability was calculated in accordance with the following equation:


(6) $$\begin{array}{r} Cell\;viability\;\left(\%\right)\;=\;\frac{A_{sample}-A_{blank}}{A_{control}-A_{blank}}\;\times \;100 \end{array}$$

where A_sample_ is the average absorbance value of the solution with different concentrations of lycopene-loaded PEC NP samples at 570 nm. A_blank_ is the average absorbance value of the solution without cell samples at 570 nm. A_control_ is the average absorbance value of the solution without lycopene-loaded PEC NP samples at 570 nm.

#### Hoechst 33342 Staining

The protective effects of lycopene-loaded PEC NPs were confirmed by performing microscopic analysis through Hoechst 33342 staining for nuclei in L02 cells in accordance with a previously published method (34).

#### Intracellular ROS Detection

As previously described by Dong et al. (34), 2,7-dichlorodi-hydrofluorescein diacetate was used to evaluate intracellular ROS generation in L02 cells, and a quadrant investigation was performed utilising WinMDI.

#### Determination of Biochemical Parameters

The activities of SOD, GSH-px, and the level of MDA were measured using commercially available kits according to the manufacturer's instructions. In brief, the L02 cells were treated with lycopene-loaded PEC NP solution for 24 h after treatment with H_2_O_2_ for 4 h. The cells were lysed; cell supernatants were collected, and MDA, GSH-px, and SOD were determined using a commercial detection kit.

#### Western Blot Analysis

Western blot analysis was performed in accordance with a previously described method with minor modification (34). Cells were treated with lycopene-loaded PEC NPs (0, 1, 5, and 10 μmol/L) for 24 h, and then, the protein expression of heme oxygenase-1 (*HO-1*), nuclear factor erythroid 2-related factor 2 (Nrf2), phospho-Nrf2 (p-Nrf2), AKT, and p-AKT were determined by Western blot analysis. Meanwhile, cells were pre-treated with 10 μM of ZnPP-IX or LY294002 for 1 h prior to incubation with or without 5 μmol/L of lycopene-loaded PEC NPs for 24 h, and then, the protein expression was determined by Western blot analysis.

### Statistical Analysis

Data were presented as the mean ± standard derivations (SDs) of three replicates. Statistical analysis was performed by analysis of variance using the OriginPro Software Version 8 (OriginLab Corp., Northhampton, MA, USA). Differences were considered statistically significant at *p* < 0.05.

## Results and Discussion

### Formation and Characterisation of PEC NPs

**Figure 3:**
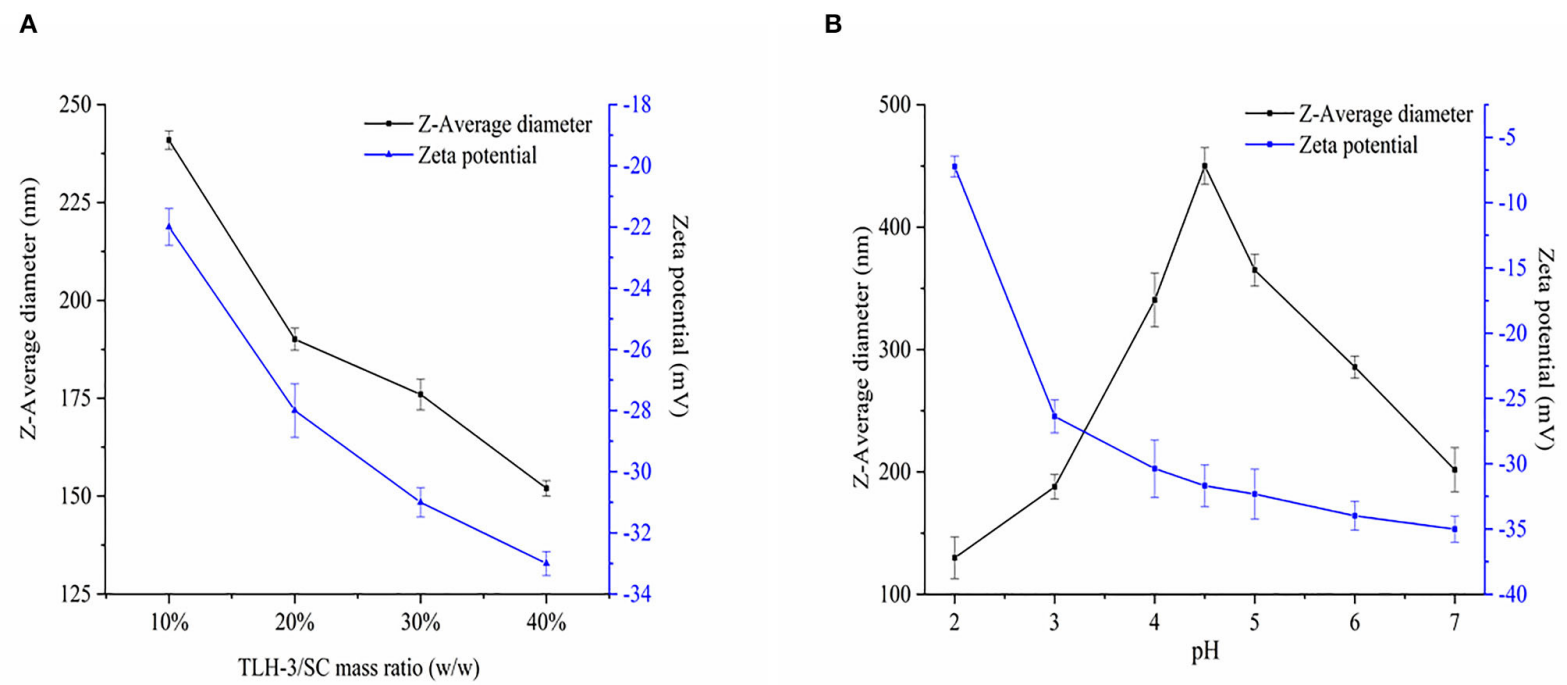
Effects of TLH-3/SC mass ratio **(A)** and pH **(B)** on the Z-average diameter, and Zeta potential of TLH-3/SC PEC NPs. Data represent the mean ± standard deviation (SD, *n* = 3).

The polysaccharide TLH-3 is an acidic one that has a molecular weight of 4.22 × 10^3^ Da, exists as a negatively charged polyanion in an aqueous solution, and forms a natural polysaccharide hydrosol. Given that SC is positively charged, TLH-3/SC PEC NPs can be formed by electrostatic interactions when TLH-3 and SC are fully hydrated. Figure 3A indicates the effect of TLH-3/SC mass ratio (10, 20, 30, and 40%) on the Z-average diameter and zeta potential of PEC NPs. TLH-3 is highly soluble in water because it contains a mass of hydrophilic carboxylic and hydroxyl groups. Therefore, the large amount of TLH-3 on the PEC NP surface is beneficial to improving the water solubility of TLH-3/SC PEC NPs. The Z-average diameter decreased from 237 nm to 150 nm when the TLH-3/SC mass ratio was increased from 10 to 40%, suggesting that the PEC NPs with a high TLH-3/SC mass ratio exhibited a small Z-average diameter. At low concentrations (such as when the TLH-3/SC mass ratio was 10%), TLH-3 cannot completely cover the SC surface. One TLH-3 molecule will be adsorbed on multiple SC molecules, thus destroying the repulsion stability of the original SC system and leading to the bridging flocculation of SC. Therefore, the resulting PEC NPs showed the largest Z-average diameter (237 nm). With further increase in the TLH-3 content, sufficient TLH-3 will be adsorbed on the SC surface and cover the SC, preventing the interconnection of multiple SC molecules and forming water-soluble TLH-3/SC PEC NPs, thus enhancing electrostatic repulsion and steric hindrance, inhibiting molecular aggregation, reducing *Z*-average diameter, and improving the stability of PEC NPs (35, 36). In an aqueous medium, the negative charge of TLH-3/SC PEC NPs significantly reduced with the increasing SC content due to the self-association of SC and the charge neutralisation of PEC NPs. However, when the mass ratio was changed from 10 to 40%, the zeta potential of PEC NPs increased from −21 mV to −33 mV because the increase in TLH-3 content also increased the negative charges on the PEC NP surface. Therefore, the obtained PEC NPs had a high net negative charge and were stable to flocculation, which was in accordance with previous studies (37, 38).

Figure 3B shows the effect of pH on *Z*-average diameter and zeta potential. When the solution pH value increased, the *Z*-average diameter of PEC NPs first increased and then decreased. When the pH was around 4.5, the *Z*-average diameter reached the maximum value. Given that this pH was close to the isoelectric point of SC, the intermolecular repulsive force decreased or even disappeared, and the *Z*-average diameter increased owing to SC coalescence or flocculation SC under the influence of van der Waals force and other interactions (39, 40). When the pH value was far from the isoelectric point of SC, the SC returned to a soluble state and the *Z*-average diameter decreased. With the increase in pH, the negative zeta potential of PEC NPs continued to increase from −7.23 to −35 mV, suggesting the electrostatic adsorption of anion polysaccharide (TLH-3) and cationic protein (SC) (16, 39). SC was positively charged when the pH was lower than the isoelectric point of SC (*IP* = 4.5) but was negatively charged when the pH was above 4.5. The zeta potential of PEC NPs rapidly increased from −7.23 to −31.68 mV within a low pH range (pH = 2–4.5) due to the increased negative charges on the PEC NP surface. At high pH (pH = 4.5–7), the change in the zeta potential value of PEC NPs was relatively small because SC still had a partial positive charge on its surface despite being negatively charged at this time. Therefore, anionic polysaccharides (TLH-3) could be absorbed onto the SC surface by local electrostatic attraction to form a weak reversible electrostatic complex, thus partially reducing the zeta potential of PEC NPs. These behaviours were consistent with the results from previous studies (16, 41).

### Characterisation of Lycopene-Loaded PEC NPs

Lycopene was encapsulated inside the PEC NPs to form lycopene-loaded PEC NPs with improved stability and bioavailability. The results of the DLS test showed that the Z-average diameter increased significantly from 190 nm to 350 nm after encapsulation, indicating that lycopene was successfully encapsulated inside the PEC NPs. The zeta potential of PEC NPs and lycopene-loaded PEC NPs were about −27.8 and −23.6 mV, respectively, indicating their stability in an aqueous solution. Furthermore, 12% lycopene was loaded into PEC NPs with a 20% TLH-3 and SC mass ratio. The final EE and LC of lycopene-loaded PEC NPs were 93.6 and 10.03%, respectively. Previous studies had reported that the encapsulation efficiency of lycopene by whey protein isolate was 64.7% (4), and the maximum encapsulation efficiency was 63.73% when plant and dairy protein blends were used as delivery vehicles for lycopene (8). Therefore, the TLH-3/SC PEC NP delivery system is superior to other similar delivery systems because of its relatively high encapsulation efficiency for lycopene, thus meeting the requirements of the food industry for nutrient delivery.

**Figure 4:**
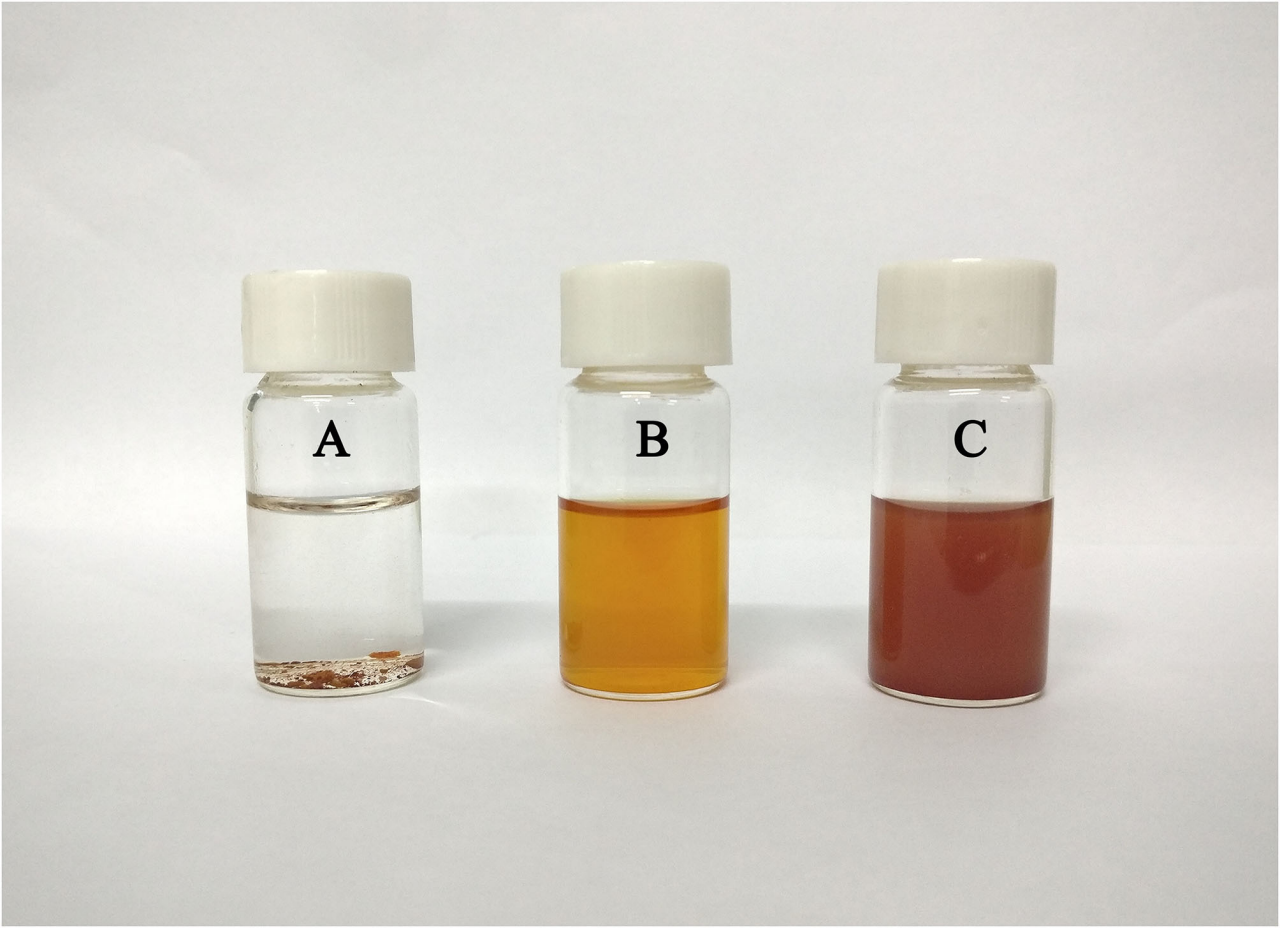
Solubility of lycopene: **(A)** lycopene in distilled water, **(B)** lycopene in acetone, and **(C)** lycopene-loaded PEC NPs in distilled water.

Free lycopene is almost insoluble in an aqueous solution due to its strong hydrophobicity and thus is deposited at the bottom (Figure 4A). In this study, lycopene was completely dissolved in acetone, and the colour of the solution was light yellow (Figure 4B). Lycopene-loaded PEC NPs were almost completely dissolved in an aqueous solution and formed a red emulsion solution (Figure 4C). These results indicated that the water solubility of lycopene was significantly enhanced after being encapsulated inside the PEC NPs.

**Figure 5:**
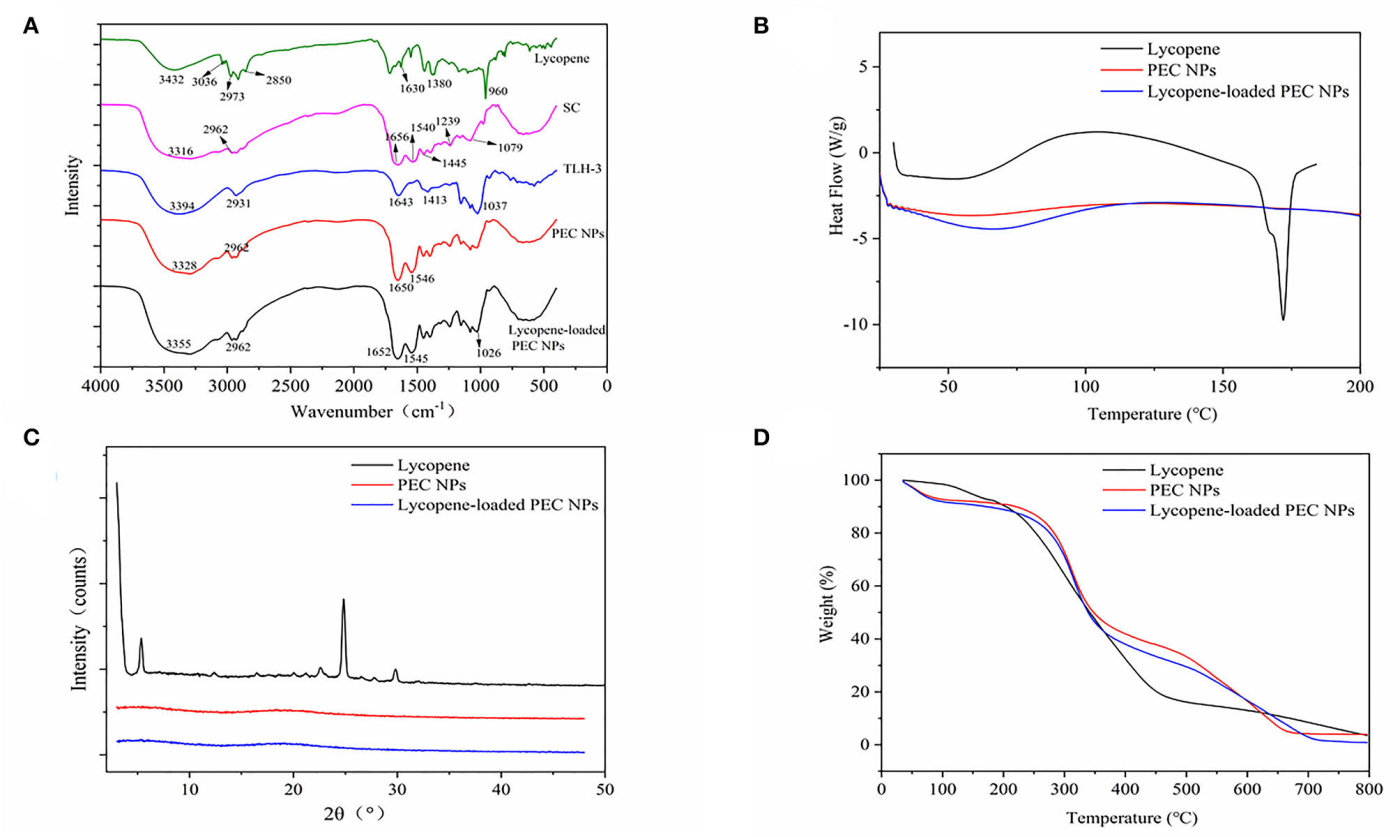
**(A)** Fourier transform infrared spectroscopy spectrum. **(B)** The differential scanning calorimetry. **(C)** X-ray diffraction spectra and **(D)** Thermogravimetric analysis.

Figure 5A shows the FT–IR spectra of lycopene, SC, TLH-3, PEC NPs, and lycopene-loaded PEC NPs. A relatively broad and strong peak (1,500–1,700 cm^−1^) was formed, indicating that the positively charged amino group of SC, namely, –NH3+ bending vibrations (1,537 cm^−1^) and C=O stretching vibrations (1,656 cm^−1^), interacted with the negatively charged COO– of TLH-3 (1,645 cm^−1^). The FT–IR spectrum also indicated that SC and TLH-3 could form relatively stable PEC NPs through electrostatic interactions rather than simple physical mixing. The characteristic absorption peaks in the lycopene spectrum at around 3036, 2973, and 2,850 cm^−1^ corresponded to C–H stretching vibrations, asymmetric methyl vibrations, and stretching vibration peaks of methyl and methylene, respectively. In addition, the absorption bands at about 1,630, 1,380, and 960 cm^−1^ were due to the C=C stretching vibrations, methyl group bending vibrations, and R1HC=CR2H (trans) rocking vibrations, respectively, which were discharged from the trans-monoenes. By contrast, some characteristic absorption peaks of lycopene vanished in the spectrum of lycopene-loaded PEC NPs, suggesting that lycopene was encapsulated inside the PEC NPs through electrostatic interaction (42). Figure 5B shows the DSC thermograms of lycopene, PEC NPs, and lycopene-loaded PEC NPs. The thermograms of lycopene showed a strong endothermic peak at about 174°C due to lycopene crystal melting, thus further illustrating the high crystallinity of lycopene. However, no endothermic peak was shown by lycopene-loaded PEC NPs before 200°C and after encapsulation. This finding confirmed the loss of the lycopene crystalline structure, suggesting that the lycopene was successfully encapsulated and formed an amorphous state. Figure 5C illustrates the XRD diffractograms of lycopene, PEC NPs, and lycopene-loaded PEC NPs. Lycopene exhibited multiple characteristic peaks (5.3, 12.4, 22.5, 24.8, and 29.8°) at 2θ from 5to 50° due to its crystalline nature. By contrast, lycopene-loaded PEC NPs displayed no characteristic peaks, suggesting that lycopene was successfully encapsulated inside the PEC NPs. A thermogravimetric analyzer was used to evaluate the TGA properties of free lycopene, PEC NPs, and lycopene-loaded PEC NPs (Figure 5D). In the second weightlessness region, the thermal decomposition temperature increased from 174°C in lycopene to 250°C in lycopene-loaded PEC NPs, suggesting that the degradation degree of lycopene-loaded PEC NPs was substantially delayed when lycopene was successfully encapsulated inside the PEC NPs. These results indicated that the thermal stability of lycopene was significantly improved after encapsulation.

**Figure 6:**
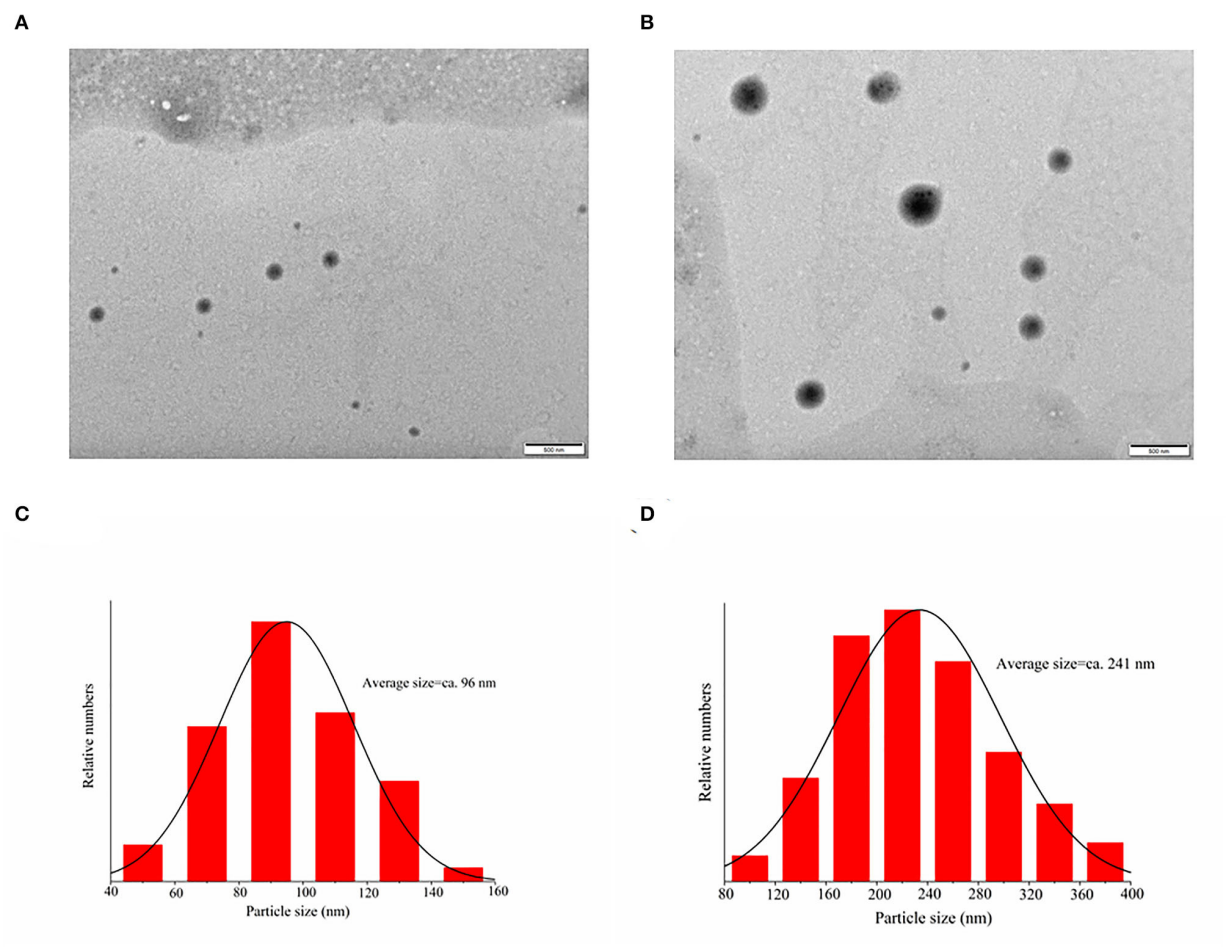
Transmission electron microscopy images of **(A)** PEC NPs; **(C)** lycopene-loaded PEC NPs; **(B)** and **(D)** are the size distribution histograms of images.

Figure 6 shows the TEM images of PEC NPs and lycopene-loaded PEC NPs. The lycopene-loaded PEC NPs (Figure 6C) exhibited a larger nanoscale spherical morphology with a smooth, uniform, and compact surface compared with the PEC NPs (Figure 6A). TEM analysis revealed that the average particle size increased from 96 nm in PEC NPs (Figure 6B) to 241 nm in lycopene-loaded PEC NPs (Figure 6D), suggesting that lycopene was successfully loaded inside the PEC NPs on a nanometre scale. This finding coincided with the greater Z-average size of lycopene-loaded PEC NPs than that of PEC NPs. In addition, the average diameter of PEC NPs (96 nm) and lycopene-loaded PEC NPs (241 nm) observed by TEM was significantly smaller than the average particle size measured by DLS (about 190 and 350 nm). This difference can be attributed to the method used: TEM describes the actual size of the sample (dry sample state), and DLS shows the hydrodynamic diameter (hydration state) of the sample. In addition, the nanoparticles display a large hydrodynamic volume in the hydration state due to solvent action (43, 44).

### Storage Stability

**Figure 7:**
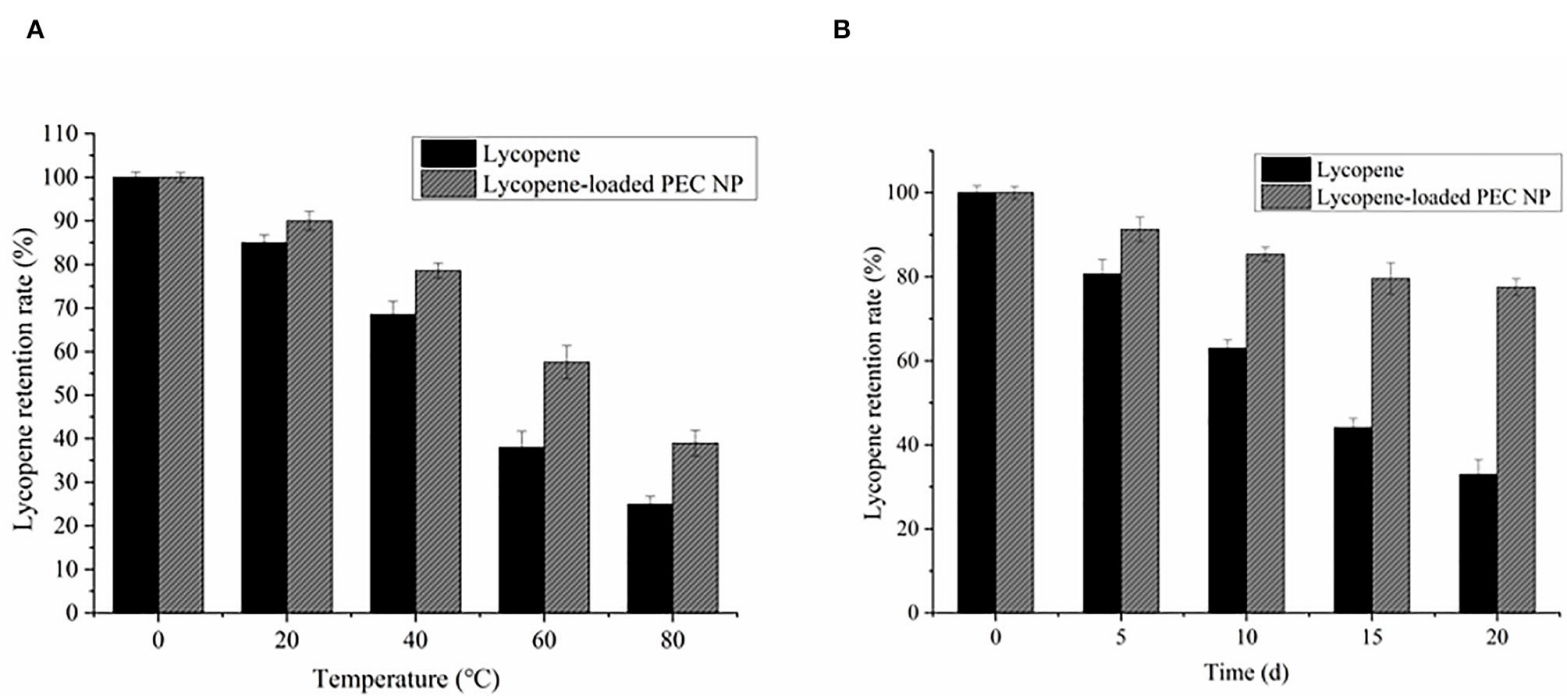
Effects of **(A)** thermal treatment **(B)** light radiation on the retention rate of lycopene and lycopene-loaded PEC NPs. Data represent the mean ± standard deviation (SD, *n* = 3).

The effects of thermal and light treatments on the lycopene encapsulated inside the PEC NPs were examined by evaluating the changes in the lycopene retention rate during the storage period (Figure 7). The thermal stabilities of lycopene encapsulated inside the PEC NPs and free lycopene were examined after incubation at different storage temperatures (0, 25, 45, 65, and 85°C) and are shown in Figure 7A. The free lycopene and encapsulated lycopene exhibited no significant differences in lycopene retention rate at low temperatures (0 °C and 25°C). However, the retention rates of free lycopene and encapsulated lycopene were approximately 37.98 and 57.55%, respectively, after heat treatment at 65°C for 0.5 h and 24.93 and 38.99%, respectively, after being heated at 85°C for 0.5 h. These results confirmed that the lycopene encapsulated inside the PEC NPs had higher retention rates than the non-encapsulated lycopene at different storage temperatures (25, 45, 65, and 85°C), thus revealing the significant improved thermal stability of lycopene. This improvement can be attributed to the spherical structure of PEC NPs being promoted and compacted by TLH-3 and SC through electrostatic interaction, thus protecting lycopene against thermal treatment. DSC, XRD, and TGA analysis results also indicated that the thermal stability of lycopene was significantly improved after encapsulation.

As illustrated in Figure 7B, the lycopene retention rate decreased with the increase in storage days. The degradation rate of encapsulated lycopene was significantly slower than that of free lycopene. The lycopene-loaded PEC NPs had a lycopene retention rate of 77.55% after 20 days of light radiation. These results suggested that PEC NPs have a good protective effect against the degradation of encapsulated lycopene under prolonged light radiation exposure from 0 to 20 days. The compact spherical structure of PEC NPs formed by TLH-3 and SC through electrostatic interaction may have protected lycopene from light radiation. Similar findings have been previously reported in previous studies, such as the improved photostability of hydrophobic nutraceuticals encapsulated in PEC NPs or ZNPs coated with biopolymers (30, 45), which revealed that encapsulating lycopene inside PEC NPs enhances its thermostability and light stability. Moreover, it was reported that the lycopene-loaded W/O emulsions were prepared by orange oil, tributyrin, and corn oil, and the corn oil lycopene emulsions were physically more stable than orange oil and tributyrin lycopene emulsions (46). Because the addition of corn oil enhanced the physical stability of the beverage during chilled storage by inhibiting Ostwald ripening, lycopene nanoemulsions were fabricated using high-pressure homogenization and using medium-chain triglycerides (MCT) as carrier oils (47). It was found that the molecules stretched into the O/W interface as the lycopene loading increased, strengthening the lateral packing of OSA molecules on the interfacial membranes and then decreasing the mean particle diameters and improving the stability of nanoemulsions.

### *In vitro* Release and Antioxidation Activities

**Figure 8:**
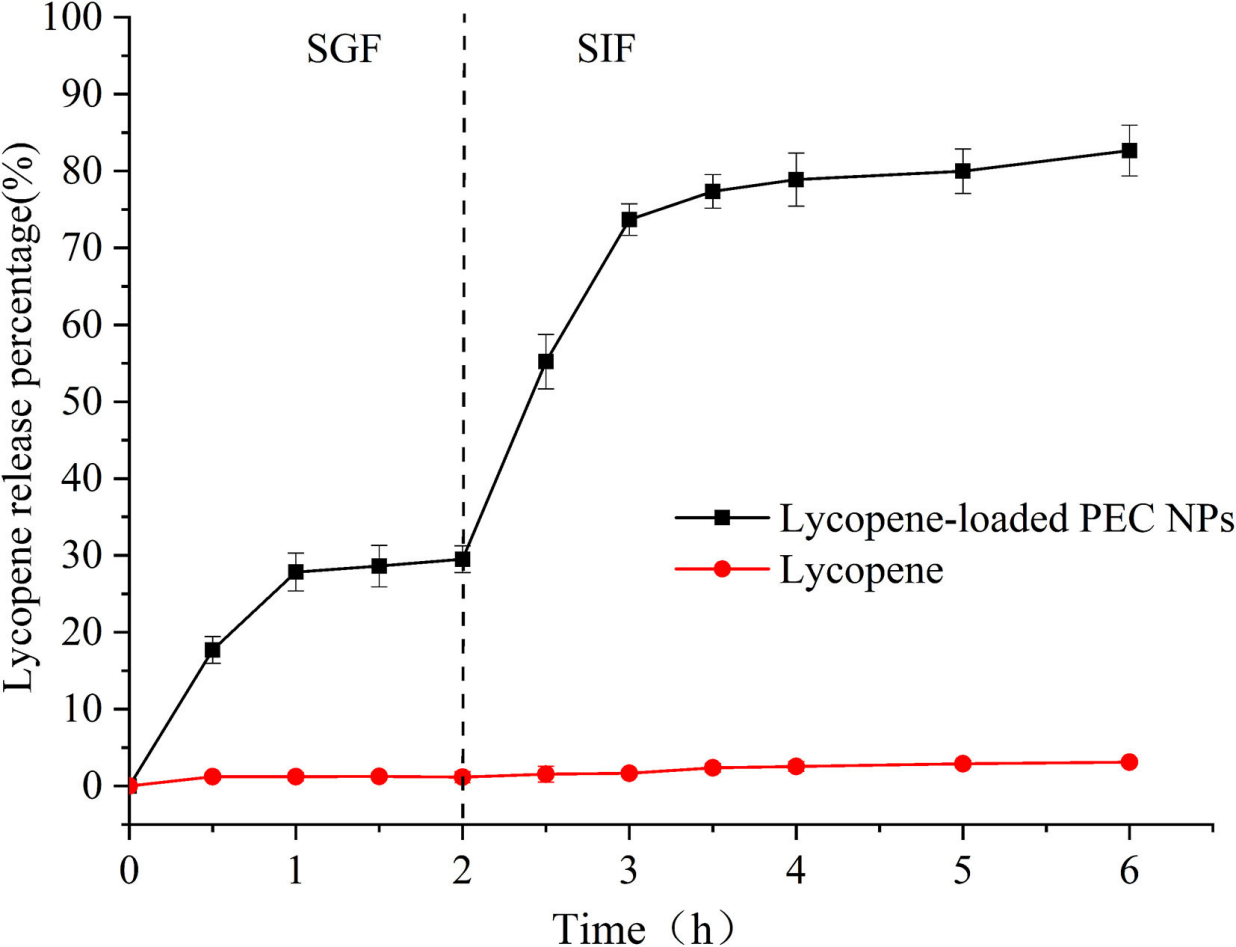
Release of lycopene in lycopene-loaded PEC NPs and free lycopene in SGF and SIF.

Figure 8 shows the *in vitro* release profiles of free lycopene and lycopene encapsulated inside PEC NPs under simulated gastrointestinal conditions. Owing to its poor water solubility, free lycopene showed negligible release rates during the simulated gastrointestinal digestion, including SGF and SIF. The lycopene encapsulated inside the PEC NPs was discharged into the SGF environment and exhibited burst release in the first 30 min of gastric digestion due to either the entrapment of lycopene near the interface or the absorption of lycopene on the PEC NP surface. Afterwards, a slight and steady release of lycopene occurred between 30 and 120 min from the start of SGF digestion. The cumulative release rates of lycopene from PEC NPs were approximately 30.15% after 2 h of SGF digestion and 85.67% after 4 h of SIF digestion, indicating that the release of lycopene was faster in SIF digestion than in SGF digestion. As indicated in 3.1, TLH-3 wrapped the hydrophobic amino acids of SC into a hydrophobic core, leading to a significant steric hindrance effect. These results suggested that the PEC NPs formed by the electrostatic adsorption of SC and TLH-3 possess a close and heavy protective layer that can increase the charge density on the PEC NP surface and the electrostatic repulsion among PEC NPs. Owing to the physical barrier formed by strong electrostatic interactions, SGF cannot directly interact with SC, which in turn could not be degraded by pepsin. Therefore, the amount of lycopene released in simulated gastric juice between 30 and 120 min was relatively small. In the simulated intestinal fluid, the electrostatic attraction of SC and TLH-3 and the TLH-3 protective layer wrapping the SC outer layer were weakened. Afterwards, SC was broken down by trypsin in SIF, and lycopene was then released from PEC NPs in large quantities. Therefore, TLH-3/SC PEC NPs significantly improved the *in vitro* control release of lycopene in the simulated gastrointestinal tract, especially burst releases in SIF. Gan et al. also reported that the bioaccessibility of phytosterol encapsulated sodium caseinate (NaCas)/pectin-based phytosterols (NCP-PSs) nanoparticles was increased by at least 43.8% compared to free phytosterols, indicating that the presence of pectin could be adsorbed on the casein micelles by electrostatic interaction and form coating, protecting the nanoparticles from degradation in the gastric environment (48). A type of lycopene nanoscale liposome carriers (NLCs) was prepared and the adsorption of NLCs in the gastrointestinal wall can prolong the contact time of lycopene with intestinal epithelial cells, thereby increasing the bioavailability of lycopene (49). Other research illustrated that sodium caseinate and pectin might protect phytosterols from degradation in the gastric environment (50). Dai et al. also reported the great improvement of curcumin release in the gastrointestinal tract environment caused by zein and rhamnolipid complex nanoparticles (44).

**Figure 9:**
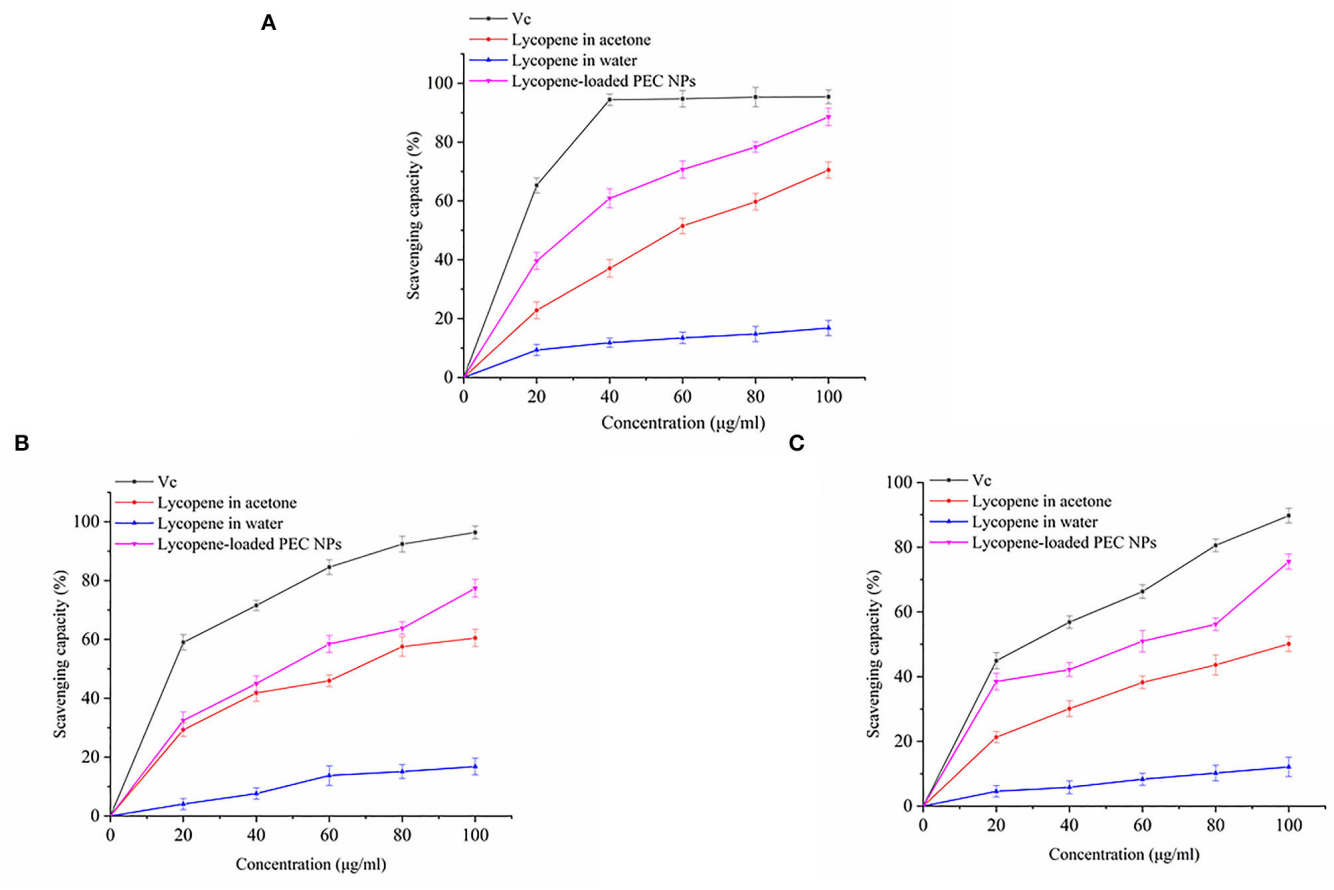
**(A)** DPPH radical scavenging activity, **(B)** Hydroxyl radical scavenging activity, and **(C)** ABTS free radical scavenging activity of Vc, lycopene in acetone, lycopene in water, lycopene-loaded PEC NPs.

The *in vitro* antioxidant activities of lycopene-loaded PEC NPs, lycopene in acetone, and lycopene in water were determined via DPPH scavenging, hydroxyl radical scavenging, and ABTS radical scavenging assays. Vc was used as the positive control. The stable DPPH radical model is a widely used method for evaluating the free radical scavenging ability of antioxidants. Figure 9A shows that, when the lycopene concentration was 0–100 μg/mL, the DPPH radical scavenging activity of the lycopene-loaded PEC NPs was higher than that of lycopene in acetone and was significantly stronger than that of lycopene in water in a concentration-dependent manner. At 100 μg/mL, lycopene-loaded PEC NPs exhibited a scavenging ability of 88.57%, which was close to that of Vc (95.41%). As the most reactive oxygen species, hydroxyl radicals can react with biological molecules and induce severe damage in living cells (51). Figure 9B shows that, after lycopene was encapsulated by SC/TLH-3 PEC NPs, the scavenging ability for the hydroxyl radical increased in a dose-dependent manner. At 0–100 μg/mL, the scavenging ability was close to that of lycopene in acetone and higher than that of lycopene in water. At 100 μg/mL, the highest scavenging ability of lycopene-loaded PEC NPs on hydroxyl radical was 77.38%, which was weaker than that of Vc (96.37%). Figure 9C indicates that the ABTS radical scavenging activity of lycopene-loaded PEC NPs at 0–20 μg/mL was close to that of Vc and at 0–100 μg/mL was always higher than that of lycopene in acetone and lycopene in water. At 100 μg/mL, the lycopene-loaded PEC NPs exhibited the highest scavenging ability of 75.53%, which was close to that of Vc (89.77%). The results showed that lycopene-loaded PEC NPs exhibited strong antioxidant activities *in vitro* that were positively correlated with their concentration. At 0–100 μg/mL, the activities of lycopene-loaded PEC NPs were higher than those of lycopene in acetone and water, indicating that the water solubility and dispersibility of lycopene were improved after being encapsulated inside the PEC NPs. As a result, its antioxidant activities were promoted. Our previous study also proved that TLH-3 exerts excellent antioxidant activities (18). The enhanced radical scavenging ability of encapsulated lycopene may be attributed to the large surface areas of PEC NPs that are beneficial to the adequate diffusion of encapsulated lycopene in the reaction medium. It can be inferred from the results that the higher the retention of lycopene in PEC NPs, the better is the antioxidant capacity of PEC NPs when compared with free lycopene. Other researchers have also reported that there is a positive correlation between the antioxidant ability and the lycopene retention of the different delivery systems (25, 52). Sodium caseinate (NaCas) is one of the milk protein components with hydrophilic and lipophilic properties that facilitate rapid absorption at the oil-water interface (53). Furthermore, proteins such as SC may have contributed to the oxidative stability of PEC NPs by forming an interfacial physical and electrostatic barrier to pro-oxidants that are common to the aqueous phase. Consequently, lycopene-loaded PEC NPs have a promising prospect in the effective prevention and treatment of diseases related to oxidative damage. Similar results have also been reported in another study, for example, curcumin encapsulated inside protein-polysaccharide nanoparticles exhibited stronger antioxidant and radical scavenging activities than curcumin solubilised in ethanol solutions (39). Lycopene was encapsulated in whey protein isolate-xylo-oligosaccharide conjugates prepared by Maillard reaction, which enhanced the emulsification performance and antioxidant capacity. Therefore, whey protein isolates glycosylated with xylo-oligosaccharides by Maillard reaction can be used to encapsulate lycopene or other bioactives and improve their properties (54). Previous research indicated that the type of carrier oil impacts the reducing power of the beverage emulsions as lycopene was more susceptible to chemical degradation in the presence of unsaturated, long-chain triglycerides, and the antioxidant capacity was reduced (46).

### Effects on Cell Viability of L02 Cells

**Figure 10:**
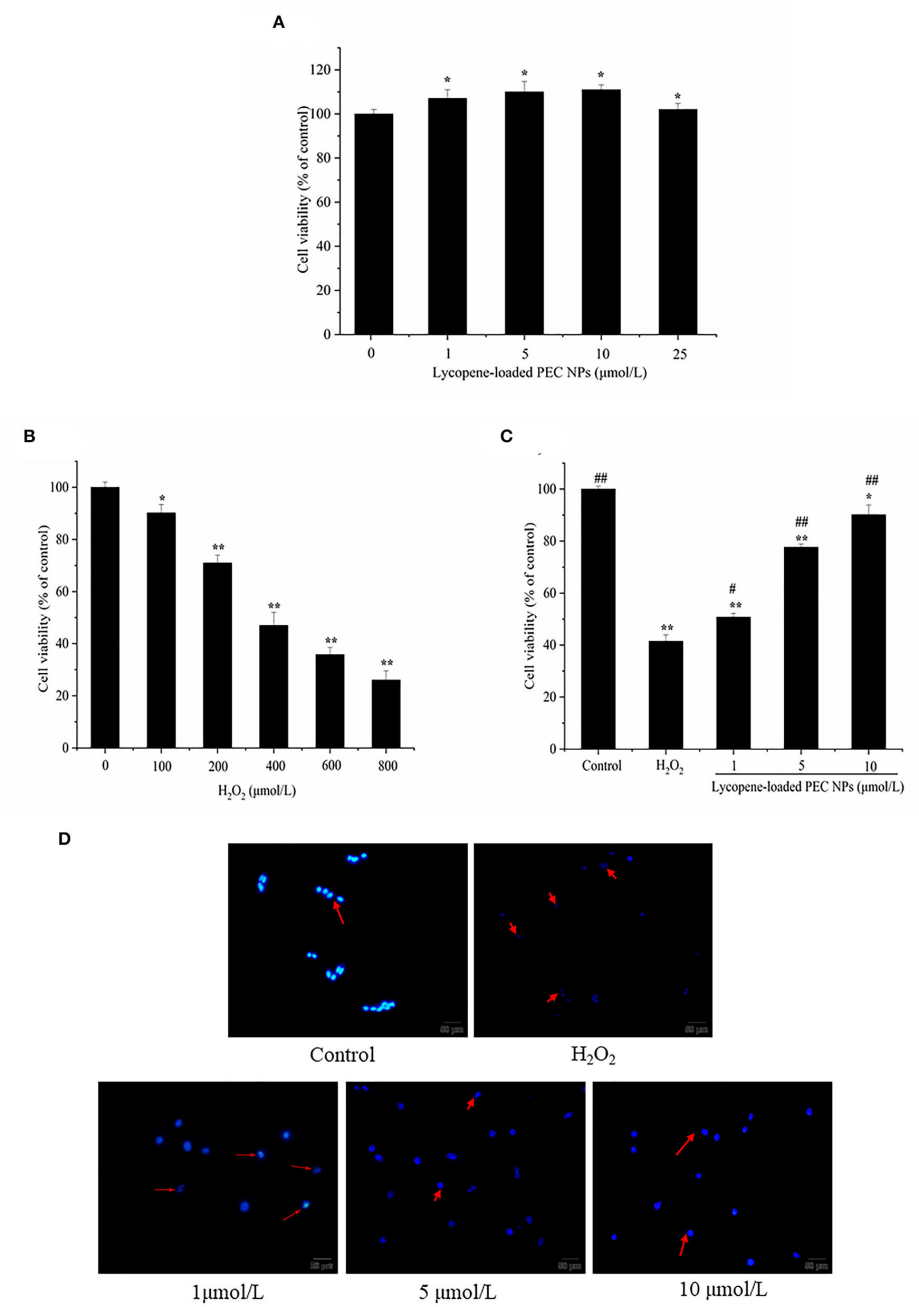
Lycopene-loaded PEC NPs inhibit H_2_O_2_-induced cell apoptosis. **(A)** Cytotoxicity of lycopene-loaded PEC NPs in cells at different concentrations. **(B)** The viability of H_2_O_2_-induced cells. **(C)** Effects of lycopene-loaded PEC NPs on the viability of L02 cells. **(D)** Morphological analysis of the nucleus in cells. Data (mean ± SD) represent three experimental replicates. * *p* < 0.05, ** *p* < 0.01, vs. control group. # *p* < 0.05, ## *p* < 0.01, vs. H_2_O_2_-treated group.

As shown in Figure 10A, lycopene-loaded PEC NPs did not cause any critical toxicity for 24 h, even at 25 μmol/L concentration. As illustrated in Figure 10B, the viability of H_2_O_2_-induced L02 cells decreased in a dose-dependent manner. Nearly 46.99% of the cells had survived when treated with H_2_O_2_ at 400 μmol/L. Hence, this concentration was used in the subsequent experiments. As shown in Figure 10C, the percentage of living cells in lycopene-loaded PEC NPs treated groups at 1, 5 and 10 μmol/L increased in a dose- and concentration-dependent manner. The H_2_O_2_-induced cells treated with lycopene-loaded PEC NPs at 10 μmol/L showed the highest percentage of living cells (90.16%). The results suggested that exposure to H_2_O_2_ resulted in an increase in cell death, but lycopene-loaded PEC NPs alleviated the oxidative damage in a dose-dependent manner and significantly protected L02 cells against H_2_O_2_-induced cell apoptosis. As a well-known antioxidant, lycopene has a protective effect on oxidative stress cell damage. For example, it was reported that lycopene decreased the apoptosis rate of H_2_O_2_-induced bovine mammary epithelial cells (bMECs) and the accumulation of intracellular reactive oxygen species (ROS) when lycopene was delivered to cells using THF as a solvent which contained 0.025% butylated hydroxytoluene (55). The hepatic cell viability decreased significantly after hypoxia/reaeration treatment but increased in a dose-dependent manner after cells were treated with different concentrations of lycopene by MTT assay (56). Previous studies revealed that nanoliposomes of lycopene (L-LYC) remarkably inhibited JNK-MAPK-induced cell apoptosis, and the total number of apoptotic cells in the ischemic cortex was significantly reduced in L-LYC pre-treatment groups when compared with the vehicle group (57). However, to our best knowledge, the protective effect of lycopene delivery systems such as PEC NPs on H_2_O_2_-induced oxidative stress cell damage has not been previously reported. The low water-solubility and gastrointestinal instability led to the low bioavailability of lycopene, thereby the higher the retention of lycopene in PEC NPs, the better the protective effect on oxidative stress cell damage when compared with free lycopene.

### Hoechst 33342 Staining

Morphological changes in L02 cells were examined with Hoechst 33342 staining to further understand the protective effects of lycopene-loaded PEC NPs (Figure 10D). The L02 cell nucleus was round and was stained homogeneously with Hoechst 33342. After H_2_O_2_ treatment, a considerable proportion of the cells displayed apoptotic characteristics with condensed and fragmented nuclei, an important marker of apoptosis. Meanwhile, the number of apoptotic cells with nuclear fragmentation was significantly reduced after treatment with lycopene-loaded PEC NPs. This finding indicated that lycopene-loaded PEC NPs can inhibit the nucleic morphological changes in H_2_O_2_-induced L02 cells. Lycopene could protect AML12 hepatic cells from apoptosis as the cells' apoptosis rate was downregulated compared with the hypoxia/reaeration injury group (56). Lycopene also ameliorated H_2_O_2_-induced SH-SY5Y cell damage and reduced the expression of apoptotic markers, such as Bcl-2, Bax, and cleaved caspase 3 (58).

### Effects on H_2_O_2_-Induced Oxidative Stress

**Figure 11:**
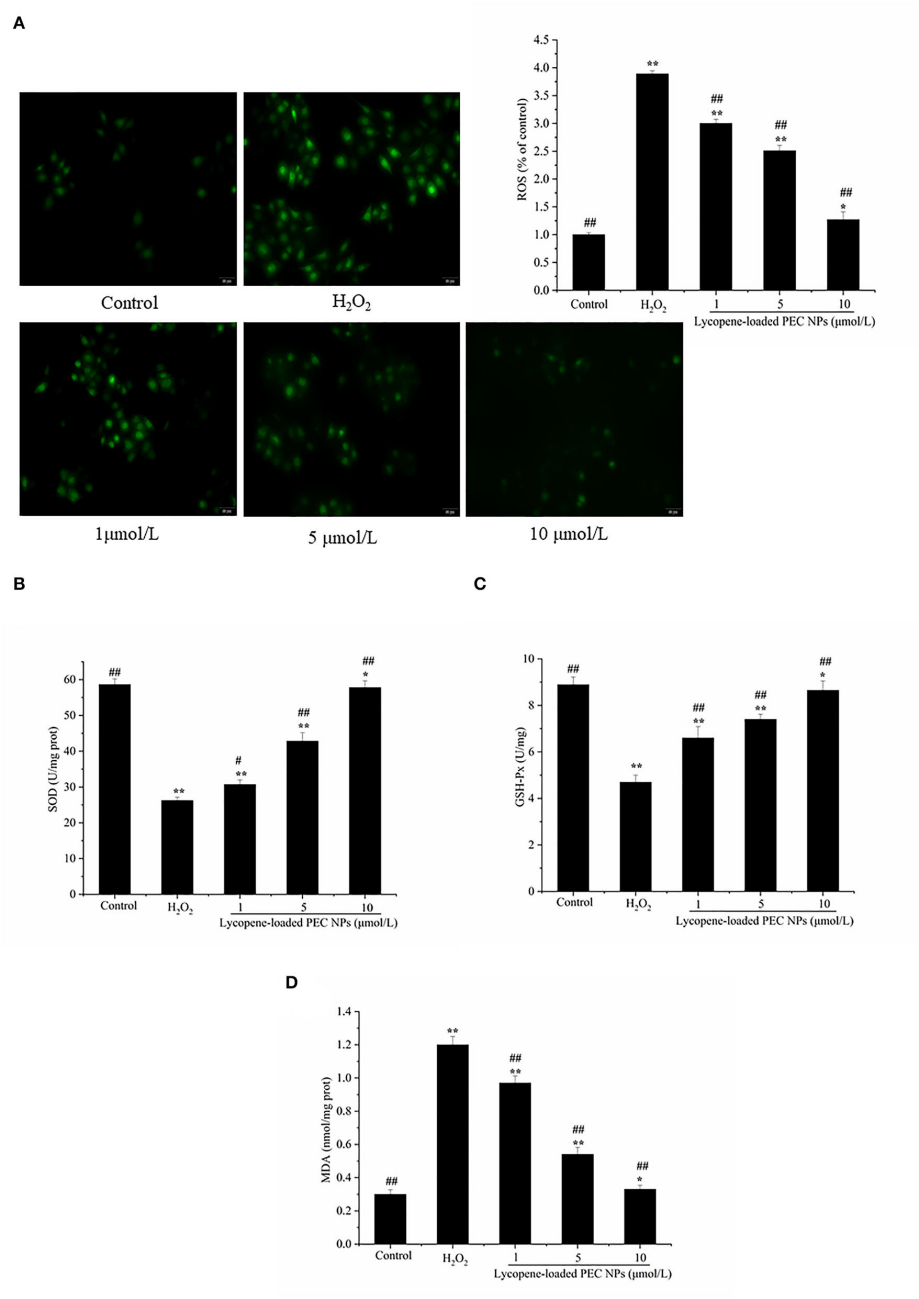
Lycopene-loaded PEC NPs alleviate H_2_O_2_-induced oxidative stress. **(A)** Effect of lycopene-loaded PEC NPs on H_2_O_2_-induced ROS generation in cells. The fluorescence increase meant that DCF was indicative of enhanced ROS generation. **(B)** Effect of lycopene-loaded PEC NPs on SOD activity. **(C)** Effect of lycopene-loaded PEC NPs on GSH-Px level. **(D)** Effect of lycopene-loaded PEC NPs on MDA level. Data (mean ± SD) represent three experimental replicates. * *p* < 0.05, ** *p* < 0.01, vs. control group. # *p* < 0.05, ## *p* < 0.01, vs. H_2_O_2_-treated group.

As shown in Figure 11A, exposure to H_2_O_2_ resulted in a 3.8-fold increase in intracellular ROS generation. Meanwhile, the L02 cells treated with lycopene-loaded PEC NPs exhibited significantly reduced ROS production, as indicated by their weak fluorescence intensity for ROS. The lycopene-loaded PEC NPs at 1, 5, and 10 μmol/L decreased the ROS generation by 21.08, 34.21, and 60.53%, respectively, compared with that in the H_2_O_2_-treated group.

As shown in Figures 11B,C, SOD and GSH-px activities were greatly reduced by H_2_O_2_ exposure but dose-dependently enhanced by treatment with lycopene-loaded PEC NPs. On the contrary, the MDA level increased after treatment with H_2_O_2_ but decreased after treatment with lycopene-loaded PEC NPs by 20.8 to 34.4% in Figure 11D. The above results suggested that lycopene-loaded PEC NPs protect against oxidative damage by stimulating antioxidative enzymes and decreasing the MDA level. Yusuf et al. deal with the encapsulation of lycopene (LYC) as polysorbate-80 (P-80) coated phosphatidylserine-chitosan self-assembled nanoparticles (P-80-LYC-PSCNP), with an approach to reduce oxidative stress and improve the antioxidant enzymatic functioning of CAT, SOD, and GPx (59). Nanoliposomes of lycopene (L-LYC) pre-treatment suppressed oxidative stress in ischemic brains, significantly elevated the total SOD, GSH, and CAT levels, and reduced the level of MDA (57).

### Protective Effects on H_2_O_2_-Treated L02 Cells

**Figure 12:**
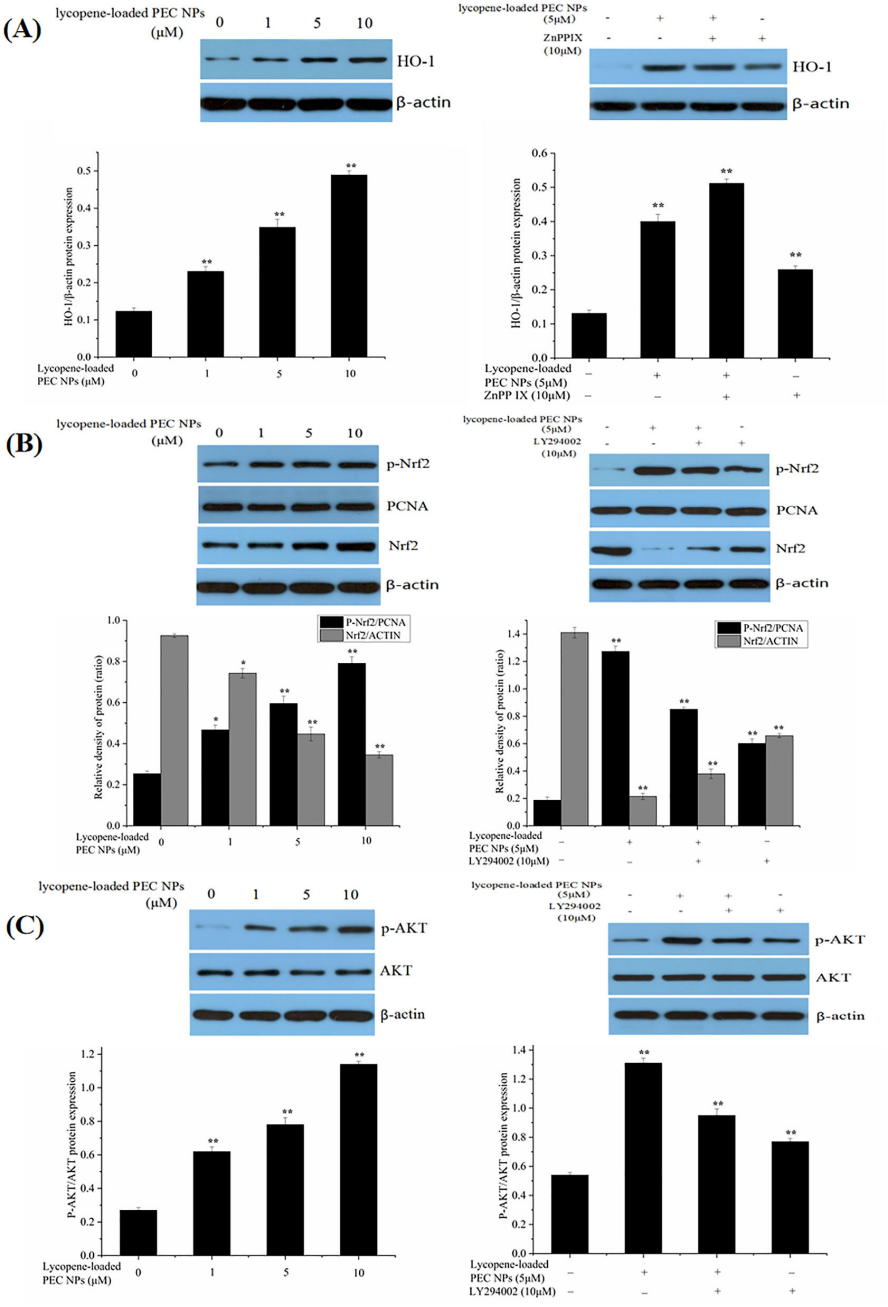
Effect of lycopene-loaded PEC NPs on protein expression of HO-1, p-Nrf2, Nrf2, p-AKT, and AKT. **(A)** Cells were treated with lycopene-loaded PEC NPs (0, 1, 5, and 10 μmol/L) for 24 h, and then protein expression of *HO-1* was determined by western blot analysis (Left); Cells were pretreated with 10 μM ZnPP-IX for 1 h prior to incubation with or without 5 μmol/L lycopene-loaded PEC NPs for 24 h and then protein expression of *HO-1* was determined by western blot analysis (Right). **(B)** Nrf2 and p-Nrf2 protein levels were measured by western blots after treatment with lycopene-loaded PEC NPs (0, 1, 5, and 10 μmol/L) for 24 h (Left); Cells were pretreated with 10 μM LY294002 for 1 h and then treated with or without 5 μmol/L lycopene-loaded PEC NPs for 24 h, and Nrf2 and p-Nrf2 were determined by western blot analysis (Right). **(C)** Cells were treated with lycopene-loaded PEC NPs (0, 1, 5, and 10 μmol/L) for 24 h, and AKT and p-AKT were determined by western blot analysis (Left); Cells were pretreated with 10 μM LY294002 for 1 h prior to incubation with or without 5 μmol/L lycopene-loaded PEC NPs for 24 h, and AKT and p-AKT were determined by western blot analysis (Right). Data (mean ± SD) represent three experimental replicates. *p < 0.05, ** *p* < 0.01, vs. control group.

The expression levels of some oxidative stress-related major genes [e.g., Nrf2, *HO-1*, and AKT] were evaluated by Western blot to determine whether lycopene-loaded PEC NPs attenuate oxidative damage in cells by inducing various signalling pathways. As indicated in Figure 12A, lycopene-loaded PEC NPs dramatically and dose-dependently enhanced the level of *HO-1* protein. However, *HO-1* expression induced by lycopene-loaded PEC NPs was suppressed after treatment with ZnPPIX, a specific *HO-1* inhibitor. As shown in Figure 12B, Western blot results indicated that the phosphorylated Nrf2 expression was remarkably increased in the groups treated with lycopene-loaded PEC NPs, suggesting that the PEC NPs significantly upregulated the phosphorylated Nrf2. Moreover, Nrf2 phosphorylation was inhibited upon pre-treatment with the special PI3K/AKT inhibitor (LY294002). These results confirmed that LY294002 could inhibit Nrf2 phosphorylation and decrease *HO-1* transcription and expression, suggesting that AKT phosphorylation is associated with Nrf2/*HO-1* signalling pathway activation. Figure 12C shows that, compared with that of the cells under normal conditions, phosphorylated AKT was significantly and dose-dependently upregulated after the cells were treated with lycopene-loaded PEC NPs. This finding suggested that pre-treatment with lycopene-loaded PEC NPs could induce AKT phosphorylation. However, a special PI3K/AKT inhibitor (LY294002) can significantly inhibit the AKT phosphorylation promoted by lycopene-loaded PEC NPs.

**Figure 13:**
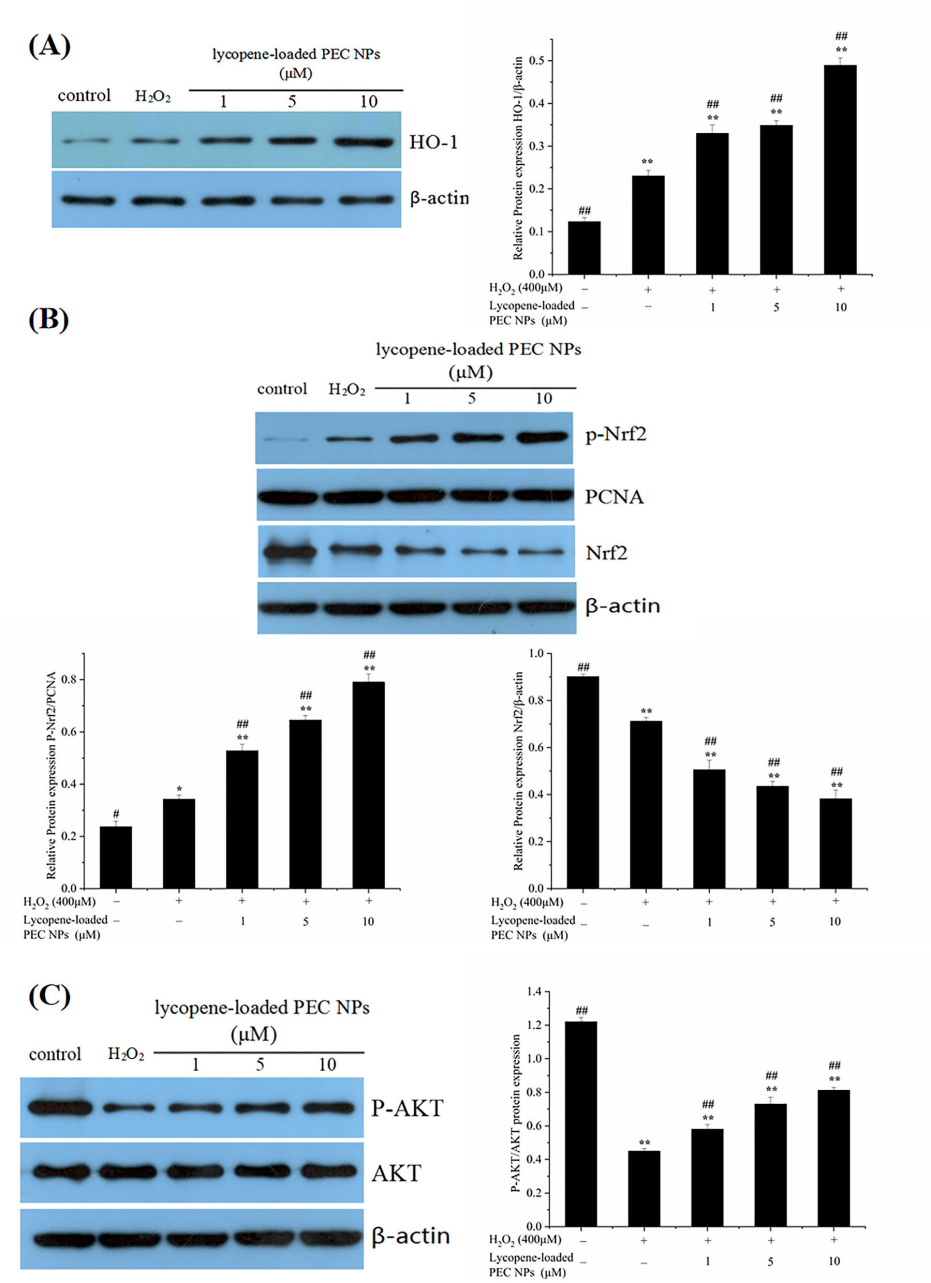
Mechanism of protective effects on H_2_O_2_-treated L02 cells. **(A)** Western blot assay of HO-1 expression; Quantification of relative protein expression quantity of *HO-1*. **(B)** Western blot assay of p-Nrf2 and Nrf2 expression. Quantification of relative protein expression quantity of p-Nrf2 and Nrf2. PCNA was used as a nuclear loading control. **(C)** Western blot assay of p-AKT and AKT; Quantification of relative protein expression quantity of p-AKT/AKT. Data (mean ± SD) represent three experimental replicates. *p < 0.05, **p < 0.01, vs. control group. ^#^*p* < 0.05, ^*##*^*p* < 0.01, vs. H_2_O_2_-treated group.

As shown in Figure 13A, *HO-1* expression levels were significantly elevated after the H_2_O_2_-induced cells were treated with lycopene-loaded PEC NPs. As illustrated in Figure 13B, phosphorylated Nrf2 was significantly and dose-dependently upregulated in the PEC NP-treated group compared with that in the H_2_O_2_ treated group, thus supporting the idea that lycopene-loaded PEC NPs could activate Nrf2 by upregulating its phosphorylation. As indicated in Figure 13C, AKT phosphorylation decreased upon exposure to H_2_O_2_ but was upregulated after treatment with lycopene-loaded PEC NPs.

Nuclear factor erythroid 2-related factor 2 is a key regulatory factor that maintains the redox balance, especially under the continuous stimulation of activated AKT (60, 61). Previous studies have confirmed that *HO-1* regulates cell apoptosis by protecting cells from oxidative damage, and its upregulation is involved in the cellular defence mechanism against oxidation (62, 63). In our study, the PI3K/AKT signalling pathway was activated by lycopene-loaded PEC NPs, thus leading to increased p-AKT levels in cells. Upon stimulation by activated AKT, Nrf2 plays an indispensable role against oxidative stress by rapidly detaching from cytoplasmic chaperone protein Keapl and transferring into the nucleus to combine with the antioxidant responsive element in the nucleus, thus transcriptionally activating phase II enzymes/antioxidant genes, such as *HO-1*, NQO1, and GCLC, and increasing the expression levels of GSH-px and SOD to maintain intracellular redox balance and remove the excess oxygen free radicals (23, 64). The above results confirmed that lycopene-loaded PEC NPs could protect L02 cells against H_2_O_2_-induced oxidative stress by activating the PI3K/AKT/Nrf2 signalling pathway and upregulating the downstream protein *HO-1*. Lycopene supplementation improves the mRNA expressions of the NQO-1 and *HO-1* as antioxidant enzymes, and lycopene decreased neuronal oxidative damage by activating Nrf2 and by inactivating NF-κB translocation in a H_2_O_2_-related SH-SY5Y cell antioxidant model (59). Lycopene also could promote the transfer of Nrf2 from the cytoplasm into the nucleus and the Nrf2/*HO-1* pathway activation, protecting hepatic cells (AML12 Cells) against hypoxia/Reaeration injury (56). Previous studies revealed that Nanoliposomes of lycopene (L-LYC) significantly reduced cerebral infarction and improved neurobehavior of the rats more efficiently than “naked” lycopene. In addition, L-LYC reduced protein levels of nitric oxide synthase and NOX2, increased the level of Bcl-2, lowered caspase-3, and suppressed apoptosis by inhibiting MAPK-JNK (56). In recent years, lycopene delivery systems have received increasing academic attention, owing to their actions in improving bioavailability (49) and treating tumours (65). However, until this moment, there are no data available about the protective effect of lycopene delivery systems, such as PEC NPs on oxidative stress-induced cell damage and the underlying molecular mechanism. Lycopene-loaded solid lipid nanoparticles (LYC-SLNs) enhanced cytotoxicity in MCF-7 breast cancer cells compared to the free lycopene, which combined with methotrexate (MTX) could be a promising approach to improve the therapeutic benefits of anticancer agents (65).

## Conclusion

**Figure 14:**
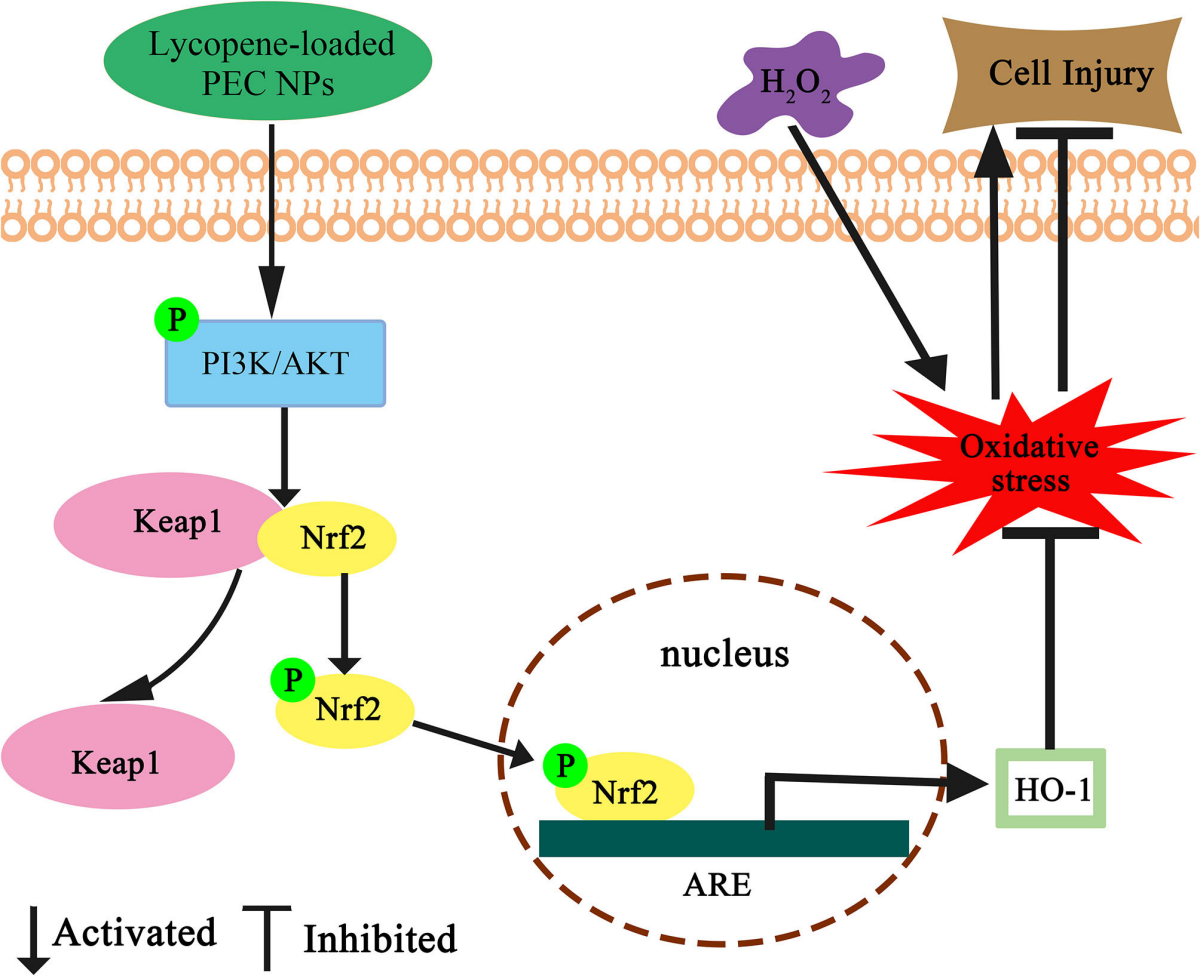
Schematic diagram for the protective effect of lycopene-loaded PEC NPs against oxidative stress in L02 cells. Where ↑ indicates increase, ↓ indicates a decrease.

Nowadays, an effective delivery system is needed to improve the absorption and bioavailability of liposoluble nutrients such as lycopene. In this study, the biocompatible TLH-3/SC PEC NPs for lycopene encapsulation were successfully constructed by electrostatic complexation. The lycopene-loaded PEC NPs with high EC and LC exhibited enhanced water-solubility, storage stability, excellent antioxidant capacity, and controlled release ability during a simulated gastrointestinal environment when compared with those of free lycopene. Furthermore, encapsulated lycopene could protect L02 cells from H_2_O_2_-induced cellular oxidative damage by reducing MDA and ROS levels, and the molecular mechanism of its antioxidation activity was summarised as the Nrf2/*HO-1*/AKT signalling pathway (Figure 14). The results indicated that the TLH-3/SC PEC NPs can be developed as effective nanocarriers for delivering liposoluble nutrients and drugs for controlled release, which has a potential application prospect in the food and pharmaceutical industries. Furthermore, this study provides a safe, green, and effective delivery system and a new idea for the development and utilisation of biocompatible PEC NPs.

## Data Availability Statement

The original contributions presented in the study are included in the article/supplementary material, further inquiries can be directed to the corresponding author/s.

## Author Contributions

XD and LC administrated the project and acquired the funding. LC and YJ conducted experiments and wrote the manuscript. DZ, MS, and MX executed the experiments and analysed the data. LC and FW reviewed and edited this manuscript. All authors contributed to the article and agreed to the published version of the manuscript.

## Funding

This work was supported by the Open Fund of State Key Laboratory of Tea Plant Biology and Utilisation (SKLTOF20190121); the Provincial Program of Natural Science of Anhui Higher Education (KJ2021A0052); Suzhou University Scientific Research Platform Open Project (2020yzd02); Anhui Quality Engineering Project (2019zyrc107); and Innovation and entrepreneurship training program for college students (202110357047).

## Conflict of Interest

The authors declare that the research was conducted in the absence of any commercial or financial relationships that could be construed as a potential conflict of interest.

## Publisher's Note

All claims expressed in this article are solely those of the authors and do not necessarily represent those of their affiliated organizations, or those of the publisher, the editors and the reviewers. Any product that may be evaluated in this article, or claim that may be made by its manufacturer, is not guaranteed or endorsed by the publisher.

## Abbreviations

PEC NPs: Polyelectrolyte complex nanoparticles
SC: sodium caseinate
ROS: Reactive oxygen species
DPPH: 2-diphenyl-1-picrylhydrazyl
ABTS, 2: 2-azinobis (3-ethylbenzothiazoline-6-sulphonic acid)
PDI: Polydispersity index
DLS: Zetasizer dynamic light scattering detector
FTIR: Fourier transform infrared
DSC: Differential scanning calorimetry
XRD: X-ray diffraction
TEM: A transmission electron microscope
SGF: Simulated gastric fluid
SIF: Simulated intestinal fluid
Vc: Vitamin C
DCFH-DA, 2: 7-Dichlorodi-hydrofluorescein diacetate
Nrf2: Nuclear factor erythroid 2-related factor 2.
